# Mitochondrial dysfunction: roles in skeletal muscle atrophy

**DOI:** 10.1186/s12967-023-04369-z

**Published:** 2023-07-26

**Authors:** Xin Chen, Yanan Ji, Ruiqi Liu, Xucheng Zhu, Kexin Wang, Xiaoming Yang, Boya Liu, Zihui Gao, Yan Huang, Yuntian Shen, Hua Liu, Hualin Sun

**Affiliations:** 1 Key Laboratory of Neuroregeneration of Jiangsu and Ministry of Education, Co-Innovation Center of Neuroregeneration, NMPA Key Laboratory for Research and Evaluation of Tissue Engineering Technology Products, Department of Neurology, Affiliated Hospital of Nantong University, Nantong University, Nantong, 226001 Jiangsu People’s Republic of China; 2 Department of Clinical Medicine, Medical College, Nantong University, Nantong, Jiangsu 226001 People’s Republic of China; 3 Department of Orthopedics, Haian Hospital of Traditional Chinese Medicine, 55 Ninghai Middle Road, Nantong, Jiangsu 226600 People’s Republic of China

**Keywords:** Mitochondrial dysfunction, Muscle atrophy, Therapy, Antioxidants

## Abstract

Mitochondria play important roles in maintaining cellular homeostasis and skeletal muscle health, and damage to mitochondria can lead to a series of pathophysiological changes. Mitochondrial dysfunction can lead to skeletal muscle atrophy, and its molecular mechanism leading to skeletal muscle atrophy is complex. Understanding the pathogenesis of mitochondrial dysfunction is useful for the prevention and treatment of skeletal muscle atrophy, and finding drugs and methods to target and modulate mitochondrial function are urgent tasks in the prevention and treatment of skeletal muscle atrophy. In this review, we first discussed the roles of normal mitochondria in skeletal muscle. Importantly, we described the effect of mitochondrial dysfunction on skeletal muscle atrophy and the molecular mechanisms involved. Furthermore, the regulatory roles of different signaling pathways (AMPK-SIRT1-PGC-1α, IGF-1-PI3K-Akt-mTOR, FoxOs, JAK-STAT3, TGF-β-Smad2/3 and NF-κB pathways, etc.) and the roles of mitochondrial factors were investigated in mitochondrial dysfunction. Next, we analyzed the manifestations of mitochondrial dysfunction in muscle atrophy caused by different diseases. Finally, we summarized the preventive and therapeutic effects of targeted regulation of mitochondrial function on skeletal muscle atrophy, including drug therapy, exercise and diet, gene therapy, stem cell therapy and physical therapy. This review is of great significance for the holistic understanding of the important role of mitochondria in skeletal muscle, which is helpful for researchers to further understanding the molecular regulatory mechanism of skeletal muscle atrophy, and has an important inspiring role for the development of therapeutic strategies for muscle atrophy targeting mitochondria in the future.

## Introduction

Skeletal muscle is a highly adaptable tissue that comprises approximately 40% of total body mass and is essential for maintaining limb posture and body movement. Skeletal muscle is also an endocrine organ that secretes myokines that have an effect on the whole-body organs [1–3]. Skeletal muscle atrophy is closely associated with some conditions such as sedentary, physical inactivity and cachexia [4, 5]. Skeletal muscle atrophy can seriously impact the quality of life of patients and increase the morbidity and mortality of many diseases. Muscle atrophy usually leads to the loss of muscle mass and function and is characterized by a reduction in muscle fiber size and mass, a conversion of muscle fiber type and an imbalance between protein synthesis and degradation in the muscle [6]. Under pathological conditions, skeletal muscle proteolysis is mainly mediated by the ubiquitin–proteasome system (UPS) and the autophagy-lysosome pathway (ALP). Muscle atrophy F-box (MAFbx) and muscle RING-finger protein-1 (MuRF1) are two muscle-specific E3 ubiquitin ligases in the UPS. During muscle atrophy, they are specifically expressed and target specific protein substrates for degradation in the UPS [7, 8]. The ALP is another important proteolysis system that removes misfolded or other harmful proteins, but its overactivation can lead to muscle atrophy [9]. Protein synthesis in skeletal muscle is mainly regulated by the IGF-1/PI3K/Akt/mTOR signaling axis, and activation of the mTOR pathway inhibits UPS and ALP in skeletal muscle [6, 10]. In addition, inflammation, oxidative stress and myofiber regeneration pathways also play a very important role in the process of muscle atrophy [11–14]. Overall, numerous molecules and complex signaling pathways have been involved in muscle atrophy; therefore, it is a major challenge for us to treat and prevent muscle atrophy.

Mitochondria are the site of ATP production and are involved in key metabolic pathways. Mitochondrial defects or dysregulation play a key role in the cytopathological mechanisms of aging, cancer, and neurodegenerative diseases [2, 15, 16]. During muscle atrophy, mitochondrial degradation influences the reduction of mitochondrial quality and quantity, which is controlled by mitochondrial autophagy as well as mitochondrial fusion and fission kinetics [17]. These mitochondrial quality-control systems are essential for the maintenance of skeletal muscle mass by recognizing and correcting mitochondrial dysfunction. Mitochondrial dysfunction triggers catabolic signaling pathways, which then will feed back to the nucleus to promote the expression of muscle atrophy genes [18]. As early as 1964, it was reported that mitochondrial dysfunction causes skeletal muscle atrophy [19]. Studies have indicated the effect of mitochondrial dysfunction on skeletal muscle atrophy. For example, in cisplatin-induced muscle atrophy, mitochondrial mass, membrane potential and reactive oxygen species (ROS) levels are abnormal. Therefore, reducing ROS production, rather than promoting ATP production, may be a therapeutic strategy to prevent cisplatin-induced muscle atrophy [20]. During muscle atrophy, denervation exacerbates mitochondrial dysregulation in muscle-specific knockout p53 tissues, suggesting that p53 promotes organ maintenance during muscle atrophy by regulating the mitochondrial quality-control process [21]. Furthermore, a clinical trial showed impaired mitochondrial function and significantly reduced level and activity of mitochondrial respiratory complex protein in pre-frail elderly (> 60 years of age) [22]. Therefore, it is an urgent task to provide an experimental basis for the treatment of muscle atrophy by targeting mitochondria.

Mitochondrial dysfunction has been recognized as an important sign of skeletal muscle atrophy, but its specific molecular mechanisms are unknown. Here, we review the role of normal mitochondria in skeletal muscle and the effects of mitochondrial dysfunction on skeletal muscle atrophy. Recent studies have also indicated that targeted modulation of mitochondrial function is an effective measure to treat and prevent skeletal muscle atrophy, which will provide an important target for developing new drugs for muscle atrophy [2, 23, 24].

## Literature search

This review focuses on a comprehensive review of the literature on mitochondria in skeletal muscle, as well as to review and analysis of the current evidences for mitochondrial dysregulation associated with skeletal muscle atrophy. The information related to the mitochondrial dysfunction were obtained from different databases and platforms, mainly PubMed, Scopus, Wiley Online Library, Springer Link, Web of Science, and Science Direct. The articles were from 1964 to 2023. After a thorough study and investigation of all the searched articles, 286 manuscripts were finally selected to complete this review based on the exclusion and inclusion criteria. More precisely, for this review, inclusion criteria included articles published in indexed and non-indexed journals, year of publication, and in vitro and in vivo investigations of mitochondria in skeletal muscle. Exclusion criteria included duplicate similar studies, poor statistical analyses, poorly written articles, poorly organized studies, and manuscripts of studies that did not meet the above inclusion criteria.

## Molecular mechanism of skeletal muscle atrophy

**Fig. 1:**
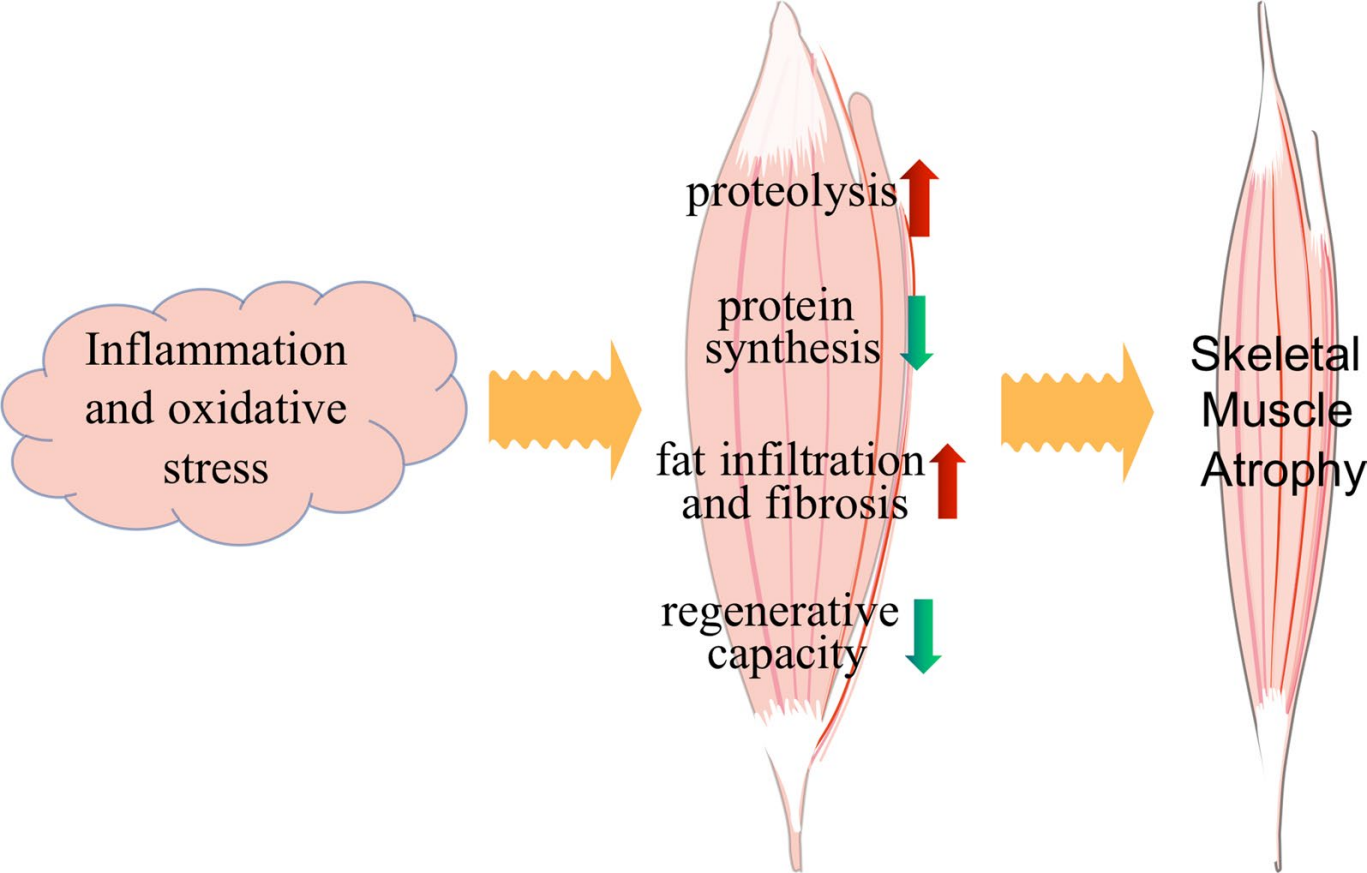
Molecular mechanism of skeletal muscle atrophy. Inflammation and oxidative stress lead to increased proteolysis, reduced protein synthesis, decreased regenerative capacity and increased fat infiltration and fibrosis

More and more studies have shown that inflammation and oxidative stress play a crucial triggering role in the process of muscle atrophy [1, 24]. Inflammation and oxidative stress lead to increased proteolysis (ubiquitin–proteasome system, autophagic-lysosomal pathway, calpain and caspase-3), reduced protein synthesis, decreased regenerative capacity and increased fat infiltration and fibrosis (Fig. 1).

### Inflammation and oxidative stress

Skeletal muscle atrophy is a state of uncontrolled inflammation and oxidative stress, which exacerbates proteolytic metabolism [25–27]. Inflammation induces skeletal muscle atrophy. During skeletal muscle injury, many key inflammatory mediators, especially inflammatory cytokines, are involved in repair process, including interferon-γ (IFN-γ), interleukin-6 (IL-6), transforming growth factor-β(TGF-β), and tumor necrosis factor-α (TNF-α) [1, 28]. Oxidative stress is a regulator of cellular signaling pathways, which influences energy metabolism, protein degradation and apoptosis in muscle through transcriptional and post-translational regulation of key proteins, leading to muscle mass loss and metabolic dysfunction [29, 30]. A transient elevation in oxidative stress levels may indicate an underlying health-promoting process, while uncontrolled accumulation of oxidative stress may have pathological implications [6, 31, 32]. Cyclooxygenase-2 (COX-2) is considered to be a positive regulator of pathophysiological processes, such as inflammation and oxidative stress, and silencing COX-2 blocks PDK1/TRAF4-induced activation of protein kinase B (AKT), which subsequently inhibits fibrogenesis after skeletal muscle atrophy [5, 33]. An anti-inflammatory drug, Triptolide, has been shown to prevent lipopolysaccharide-induced skeletal muscle atrophy by inhibiting the NF-κB/TNF-α pathway [34]. ROS, a by-product of mitochondrial metabolism, causes progressive damage to key cellular macromolecules (lipids, proteins and DNAs), and skeletal muscle is particularly susceptible due to its high metabolic rate [35]. During contractile activity, membrane-localized nicotinamide adenine dinucleotide phosphate oxidases (NAPDH) are a source of superoxide in skeletal muscle, which play an important role in redox signaling. In aging muscle, redox signaling dysregulation may lead to the loss of muscle fibers [36]. In addition, activation of toll-like receptor 2 induces oxidative stress and inflammation, while inhibition of toll-like receptor 2 attenuates skeletal muscle atrophy in a mouse model [37]. Salidroside and Tinospora cordifolia alleviate denervation-induced muscle atrophy by inhibiting oxidative stress and inflammation [12, 38, 39]. In the meantime, chronic inflammation can activate NAPDH oxidase and other inducible enzyme families that periodically promote the production of ROS and trigger further inflammation in skeletal muscle [40]. Therefore, inflammation and oxidative stress play an important regulatory role in the process of skeletal muscle atrophy, where inflammatory responses trigger oxidative stress responses and conversely, oxidative stress responses activate inflammatory responses.

### Increased proteolysis

During skeletal muscle atrophy, the main proteolysis systems include UPS, ALP, calpain and caspase-3 [6, 41–43]. The UPS is responsible for the degradation of most misfolded or defective proteins in cells, which are modified through ubiquitination, that is, covalent binding to small proteins called ubiquitins. The ubiquitination process involves three enzymes, E1 ubiquitin activating enzyme, E2 ubiquitin coupling enzyme and E3 ubiquitin ligase, ultimately leading to the rapid degradation of muscle mass [44, 45]. MAFbx and MuRF1 were the first identified E3 ubiquitin ligases that play an important role in muscle atrophy, both of which are identified as the landmarks of muscle atrophy [46, 47]. They mediate the polyubiquitination of proteins and are eventually degraded by the 26S proteasome [48]. Under the condition of aging, injury and chronic disease, UPS is significantly activated, which destroys the homeostasis of protein and causes the accumulation of protein aggregates and the imbalance of redox [1, 5, 6, 49]. The ALP is an important proteolysis system in muscle atrophy [5, 7, 33, 50]. Autophagy can be divided into chaperone-mediated autophagy, micro-autophagy and macro-autophagy (hereinafter referred to as autophagy). The ALP is mainly involved in two processes, where autophagosomes deliver cytoplasmic components that are then degraded by lysosomal hydrolases [51]. FoxO3 is a key factor in the regulation of autophagy during skeletal muscle atrophy and controls the expression of several autophagy-related genes, the most important of which is BCL2/adenovirus E1B interacting protein 3 (BNIP3) [52]. In denervated skeletal muscle, lysosomal dysfunction may limit degradation capacity, leading to an inability to clear dysfunctional mitochondria and increased ROS signaling, thus accelerating muscle atrophy [53]. Both ALP and UPS are important pathways that regulate protein degradation in muscles and complement each other to play an important regulatory role in the control of muscle mass [54]. Calpain and caspase-3 act on the upstream of UPS to aid in the complete proteolysis of myofibrillar proteins [55]. Inhibition of calpain activity prevents caspase-3 activation, and inhibition of caspase-3 activity also prevents calpain activation. There is regulatory crosstalk between these proteases, which are required for fixation-induced muscle atrophy [56]. Increased protein oxidation triggers a progressive increase in the degradation of myofibrillar proteins using calpain and caspase-3, which may link oxidative stress to the accelerated proteolysis of myofibrillar protein during disuse atrophy [57]. After aspiration pneumonia, pro-inflammatory cytokines induce muscle proteolysis through activation of calpain and caspase-3, thereby causing skeletal muscle atrophy [58]. Superoxide-mediated oxidative stress leads to overall protein degradation and accelerates skeletal muscle atrophy through the activation of UPS and ALP, accompanied by the upregulation of calpain and caspase-3 [59]. Calpain and caspase-3 act synergistically to induce skeletal muscle proteolysis, with the potential to cause oxidative stress, thereby exacerbating skeletal muscle atrophy.

### Reduced protein synthesis

Protein synthesis in skeletal muscle is a highly complex process that can be influenced by nutritional status, mechanical stimuli, repair procedures, hormones and growth factors [60]. Protein synthesis is controlled by the translation efficiency and capacity (the number of ribosomes) of mRNA into peptides [61]. Insulin-like growth factor-1 (IGF-1) is a key growth factor that regulates anabolic and catabolic pathways in skeletal muscle, and it promotes protein synthesis through the PI3K/Akt/ mTOR pathway [48]. MTORC1 activation leads to protein and lipid synthesis, and cellular growth [62]. In the skeletal muscle immobilized or disused state, mTORC1 and Akt signaling decreases, thus reducing muscle protein synthesis [63]. Hindlimb suspension-induced muscle atrophy results in increased protein synthesis and decreased protein degradation, and MAFbx and MuRF1 expression is also elevated in muscle [64]. Compared with wild-type mice, renalase-deficient mice could delay denervation-induced muscle atrophy via increased protein synthesis (Akt and p70S6K) [65]. In addition, both aerobic and resistance exercise can safely and effectively alleviate skeletal muscle atrophy by regulating myogenesis, protein synthesis and degradation, and apoptosis through the IGF-1/PI3K/Akt pathway in a mouse model of myocardial infarction [66]. Muscles play an important role in systemic protein metabolism, and promoting protein synthesis will inhibit the onset of muscle atrophy. Therefore, balancing protein synthesis and degradation is necessary to maintain a healthy state of skeletal muscle.

### Decreased regenerative capacity

Skeletal muscle is a dynamic tissue that has two unique abilities: one is the regenerative capacity, which is due to the activity of muscle satellite cells (MuSCs) unique to the skeletal muscle; the other is the adaptation of myofiber size to external stimuli, intrinsic factors or physical activity, which is known as plasticity [67]. Muscle regeneration depends on several variables, for example, MuSCs number, activation and proliferation, myogenesis and fusion all contribute to skeletal muscle regeneration [68]. The activation and differentiation processes of MuSCs are finely controlled by genetic reaction cascades involving Pax7 and myogenic regulatory factors (Myf5, MyoD, myogenin and MRF4) [69]. These factors drive each step of skeletal muscle regeneration until the formation of new myofibers [69]. Fibroadipogenic progenitors are essential for skeletal muscle regeneration and are required for the long-term homeostatic maintenance and growth of skeletal muscle [70]. It has been shown that chronic denervation leads to a reduction in muscle stem cell populations, thereby negatively influencing the regenerative ability of muscles following innervation [71]. Although MuSCs depleted mice exhibit no sign of increased muscle loss, MuSCs depletion leads to environmental disturbance of the surrounding muscle, resulting in increased fibrosis and decreased muscle mass and function [72]. Clearly, MuSCs are important in the development and regeneration of skeletal muscle. Predictably, muscle stem cell therapy will be a major challenge in skeletal muscle regeneration and repair.

### Increased fat infiltration and fibrosis

Myosteatosis (pathological fat accumulation in muscle) is defined by lower mean skeletal muscle radiodensity in computed tomography (CT) [73]. Skeletal muscle fibrosis is a common hallmark of chronic injury, such as injury caused by sarcopenia or denervation [74]. Skeletal muscle fat infiltration is associated with inflammation and fibrosis. An increase in muscle fat deposition, adipocyte infiltration and myofibrosis may play an additional role in the pathogenesis of sarcopenia in aging skeletal muscle [75–77]. TGF-β is a regulator of fibrosis and inflammation in many tissues and can trigger skeletal muscle atrophy and fibrosis by inducing atrogin-1 and scleraxis [78]. Fat infiltration into muscle and bone, as well as the redistribution of subcutaneous fat into the intra-abdominal region (visceral fat), can lead to a decrease in overall strength and function, an increase in risk of falls and fractures, and a possible increase in morbidity following sarcopenia [79]. Inhibition of Toll-like receptor 9 attenuates skeletal muscle fibrosis in aged mice with sarcopenia via the p53/SIRT1 pathway [80]. Increased secretion of complement component 1q with aging results in muscle fibrosis and atrophy, while resistance training can reduce muscle fibrosis and atrophy via downregulating the C1q-induced Wnt signaling in aging mice [81]. In addition, radiothione prevents radiation-induced muscle fibrosis by modulating Nrf2-mediated antioxidant activity and downregulating the TGF-β1/Smad pathway [82]. In summary, increased muscle fat infiltration and fibrosis are the hallmarks of muscle atrophy. Increased muscle fat infiltration and fibrosis, as well as increased proteolysis and decreased protein synthesis and regeneration, are influenced by inflammation and oxidative stress.

## Structure and function of mitochondria

Skeletal muscle is crucial for body movement, energy metabolism and substance metabolism, and directly affects the quality of human life [83]. Skeletal muscle fibers have two mitochondrial populations with different subcellular locations: subsarcolemmal mitochondria (about 20%) gather under the myolemma and intermyofibrillar mitochondria (about 80%) arrange in an order pattern between the myofibrils [84, 85]. Mitochondria have two layers of membranes (outer membrane and inner membrane) and two compartments. The two compartments are the innermost matrix and the intermembrane space between the outer membrane and inner membrane. Mitochondria are composed of about 1000 different proteins and about 400 different lipids, including fatty acids, glycerophospholipids, glycerolipids, sphingolipids and aryl alcohols [86]. Mitochondria play a central role in cellular metabolism, cell proliferation, cell death, and the redox state of the cell [87]. To maintain adequate mitochondrial homeostasis, cells have many mitochondrial quality-control processes and protective compensatory pathways that can be activated in response to a certain level of mitochondrial dysfunction [88]. Mitochondria are essential organelles that are responsible for regulating the metabolic state of skeletal muscle, and the preservation of mitochondrial structure and function is an important determinant for maintaining skeletal muscle fitness.

## Role of mitochondria in muscle atrophy

### The role of normal mitochondria in skeletal muscle

#### Mitochondrial dynamics

Mitochondria are highly dynamic organelles that undergo a coordinated cycle of fission and fusion, called ‘‘Mitochondrial dynamics.’’ Mitochondrial fission and fusion are considered to be key processes related to mitochondrial and cellular health [89]. Mitochondrial fusion and fission dynamically control the cell cycle, metabolism and survival, which are associated with a variety of physiological and pathological conditions. Mitochondrial fusion occurs in two steps, starting with the outer mitochondrial membrane fusion mediated by mitofusin 1 (MFN1) and mitofusin 2 (MFN2), followed by the inner mitochondrial membrane fusion mediated by optic atrophy 1 protein (OPA1). In contrast, mitochondrial fission produces a large number of small fragments, mainly mediated by dynamin-related protein 1 (Drp1), fission protein 1 (Fis1), mitochondrial fission factor (Mff) and mitochondrial dynamics protein of 49 and 51 (MiD49/51) [90, 91]. During the mitochondrial life cycle, fission leads to both the biogenesis of new mitochondria and the removal of dysfunctional mitochondria through mitochagy [92]. Thus, mitochondrial dynamics plays a very important role in maintaining skeletal muscle and mitochondrial integrity and function.

#### Mitochondrial biogenesis

Mitochondrial biogenesis is a process that generates new mitochondria from existing mitochondria. The biogenesis process is regulated by PPAR-gamma coactivator-1 alpha (PGC-1α). Once activated by phosphorylation or deacetylation, PGC-1α activates nuclear respiratory factor 1 and 2 (NRF1 and NRF2) and subsequently activates mitochondrial transcription factor A (TFAM) [93, 94]. Mitochondrial biogenesis is driven with three main steps: transcription of nuclear genes, import of nuclear encoded mitochondrial protein, and transcription and replication of mitochondrial DNA (mtDNA) [95]. Although the coordinated control of mitochondrial biogenesis is mainly achieved through nuclear genome-encoded factors, mitochondria-related mechanisms, such as mitochondrial protein import and mtDNA replication, transcription, and translation, are also indispensable to mitochondrial biogenesis [96]. In yeast and mammalian cells, ROS regulate mitochondrial biogenesis through the regulation of Hap4p and PGC-1α [97]. Mitochondrial biogenesis regulates the homeostasis of mitochondrial mass and function. Exploring the coordinated role of signaling pathways involved in mitochondrial biogenesis and mitochondrial dynamics should be the focus of future work.

#### Mitophagy

Autophagy is essential for muscle quality control, contributing to the degradation of damaged or aggregated proteins and the turnover of basal proteins [98]. Mitophagy is a mitochondria-specific autophagy regulated by PINK1-Parkin-dependent and PINK1-Parkin-independent pathways [99]. To specifically remove damaged or redundant mitochondria, dysfunctional mitochondria recruit PINK1 and subsequently activate parkin, leading to ubiquitination of outer membrane proteins. This mitochondrial ubiquitination complex is wrapped by autophagosomes and then degraded in the lysosome [100, 101]. Mitochondrial fission and fusion regulate their size, number, morphology and distribution in cells. Mitochondrial fission and fusion controls autophagic degradation of dysfunctional mitochondria to maintain a healthy physiological state [102]. Bcl2 has been found to be a key regulator of exercise-induced autophagy in vivo, and autophagy may contribute to the beneficial effects of exercise on metabolism [103]. Mitophagy is coordinated with mitochondrial fission and fusion to maintain the mitochondrial quality control, required for normal metabolism and growth and development of skeletal muscle.

### The role of mitochondrial dysfunction in skeletal muscle atrophy

**Fig. 2:**
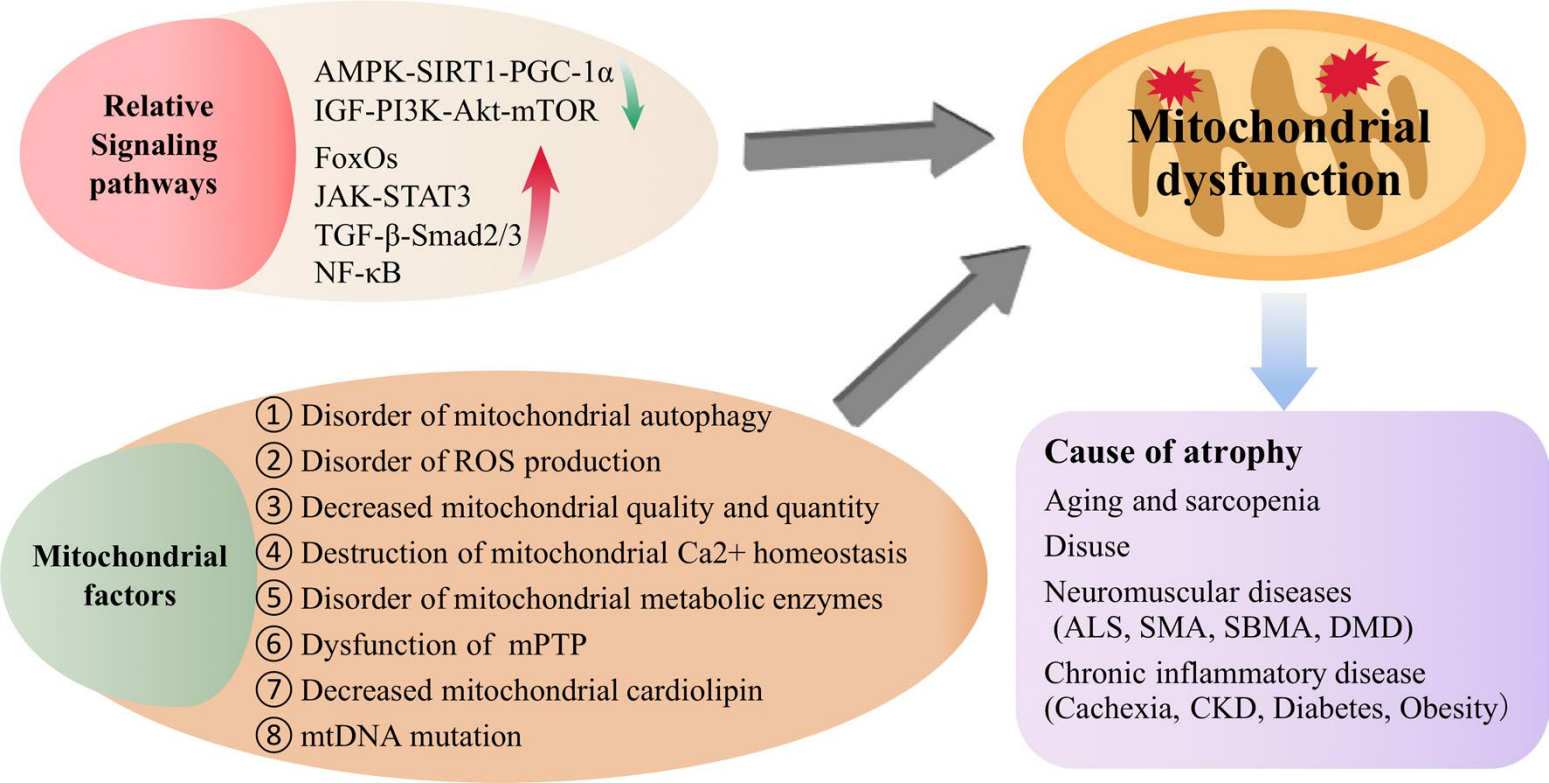
The role of mitochondrial dysfunction in skeletal muscle atrophy

Mitochondria are the key organelles regulating the metabolic state of skeletal muscle. Mitochondrial dysfunction will directly affect the normal state of skeletal muscle. In the context, we will outline the signaling pathways involved in regulating mitochondrial function and the role and mechanisms of mitochondrial factors in mitochondrial dysfunction, and discuss the role of mitochondrial dysfunction in skeletal muscle atrophy caused by different diseases, including aging and sarcopenia, disuse muscle atrophy, neuromuscular diseases and chronic inflammatory disease (Fig. 2).

#### Signaling pathways involved in mitochondrial dysfunction

**Fig. 3:**
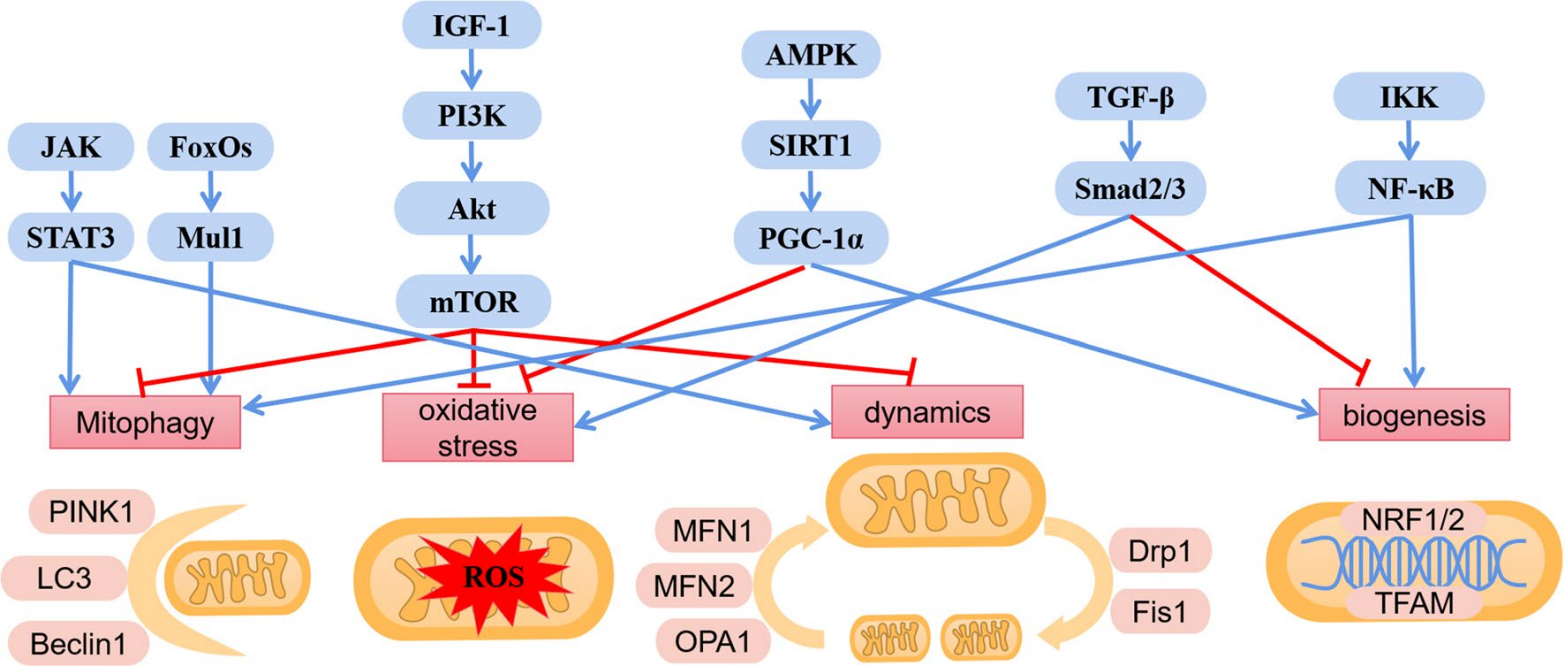
The related proteins involved in the mitochondrial dysfunction

Signaling pathways involved in mitochondrial dysfunction are very complex and includes AMPK-SIRT1-PGC-1α, IGF-1-PI3K-Akt-mTOR, FoxOs, JAK-STAT3, TGF-β-Smad2/3, NF-κB signaling pathways and so on (Fig. 3, Table 1).

**Table 1:** Roles of signaling pathways involved in regulating mitochondrial function in mitochondrial dysfunction

Signaling pathways	Roles in mitochondrial dysfunction	References
FoxOs	Activate UPS and ALP; upregulate Mul1, influence mitochondrial dynamics (increased mitochondrial fission, accelerated mitochondrial fusion protein degradation), and induce excessive autophagy	[83, 102]
AMPK/SIRT1/PGC-1α	Promote mitochondrial biosynthesis; involve in the regulation of energy metabolism-related genes; reduce oxidative stress	[103, 105, 106]
IGF-1/PI3K/Akt/mTOR	Promote complex I-driven mitochondrial respiration and supercomplex assembly; regulate the balance between mitochondrial fusion and fission; inhibit oxidative stress and autophagy	[111–114]
JAK/STAT3	Induce mitochondrial respiration; promote mitochondrial apoptosis and autophagy; increase the expression of mitochondria-related proteins (PGC-1α, OPA1, MFN2, cytochrome C)	[14, 117–119]
TGF-β/Smad2/3	Disrupt redox signaling and result in the accumulation of ROS; inhibit mitochondrial biogenesis; downregulate the abundance of PGC-1α, AMPK-α2, TFAM, and mitochondrial enzymes	[121–124]
IKK/NF-κB	Inhibit differentiation and promotes mitochondrial biogenesis; increase the expression of autophagy-related Beclin1; disrupt mitochondrial respiratory function/morphology, induce ROS production, and increase the expression of key mitochondrial genes (SDHA, ANT-1, UCP3, and MFN2)	[125, 126, 128]

##### AMPK-SIRT1-PGC-1α signaling pathway

The AMP-activated protein kinase (AMPK)/silent information regulator 1 (SIRT1)/PGC-1α signaling pathway acts as an energy sensing network that is crucial for mitochondrial biosynthesis, energy metabolism and oxidative stress [104]. AMPK is a core component of the AMPK-SIRT1-PGC-1α signaling pathway that regulates the switch between anabolic and catabolic metabolism [2, 105]. AMPK controls the expression of genes involved in energy metabolism in mouse skeletal muscle by synergizing with another metabolic sensor, SIRT1, leading to deacetylation of downstream SIRT1 targets (e.g., PGC-1α) [106]. In obesity-induced muscle atrophy, fibroblast growth factor 19 may promote mitochondrial biogenesis and antioxidant responses via the AMPK/PGC-1α pathway, thereby attenuating the effects of palmitate on mitochondrial dysfunction and oxidative stress [107]. Ampelopsin attenuates d-gal-induced skeletal muscle atrophy in aged rats by activating the AMPK/SIRT1/PGC-1α pathway [108]. Considering the AMPK-SIRT1-PGC-1α signaling pathway directly regulates mitochondrial biosynthesis, the activation of this pathway can reduce the negative effects of oxidative stress on skeletal muscle.

##### IGF-1-PI3K-Akt-mTOR signaling pathway

IGF-1-PI3K-Akt-mTOR is a well-characterized anabolic pathway that plays an important role in the regulation of protein degradation [109]. IGF-1, binding to the IGF-1 receptors on the surface of myocytes, phosphorylates insulin receptor substrate-1 (IRS1) and then activates PI3K. PI3K accelerates the adhesion of Akt to the cell membrane where it is phosphorylated. Akt then activates mTORC1 to regulate autophagy [110, 111]. Insulin-deficient diabetes or loss of insulin/IGF-1 action in muscle reduces complex I-driven mitochondrial respiration and supercomplex assembly, in part due to FoxO-mediated inhibition of complex I subunit expression [112]. The IGF-1 signaling system has been found to increase sensory protection by regulating the balance between mitochondrial fusion and fission, thereby improving the functional status of chronically denervated skeletal muscle cells [113]. During skeletal muscle hypertrophy, the relative reduction in mitochondrial function or content is complemented by enhanced mitochondrial fusion, and this complementary response may be regulated by mTORC1 [114]. In addition, butyrate ameliorates skeletal muscle atrophy in a db/db mouse model of diabetic nephropathy by enhancing activation of the PI3K/Akt/mTOR pathway to inhibit oxidative stress and autophagy [115]. Neuromodulin-1β alleviates sepsis-induced skeletal muscle atrophy in rats by inhibiting autophagy through the Akt/mTOR signaling pathway [116]. Once this pathway is dysregulated, protein synthesis, protein hydrolysis and mitochondrial function are all affected, and then the homeostasis of the skeletal muscle is bound to be disrupted.

##### FoxOs signaling pathway

FoxOs regulate the expression of atrophy-related genes (MuRF1 and MAFbx) and autophagy-related genes in skeletal muscle cells, and coordinate the two major proteolysis systems (UPS and ALP) [12, 46, 117, 118]. FoxO activates UPS and ALP simultaneously, ensuring that the loss of different cellular components such as proteins and organelles during muscle atrophy is coordinated [85]. Mitochondrial E3 ubiquitin protein ligase 1 (Mul1) is a mitochondrial ubiquitin ligase that plays an important role in the remodeling of the mitochondrial network. Under catabolic conditions, the FoxOs family upregulates Mul1, induces autophagy, and increases mitochondrial fission, leading to MFN2 ubiquitination and degradation [119]. The FoxOs family may induce mitochondrial dysfunction by triggering mitochondrial dynamics and excessive autophagy.

##### JAK-STAT3 signaling pathway

The IL-6 cytokine family activates the JAK/STAT3 signaling pathway and regulates MuSCs-dependent myogenesis [120]. Fam3a is a STAT3-regulated secretory factor, and the STAT3-Fam3a axis has been found to promote myogenic differentiation of muscle stem cells by inducing mitochondrial respiration [121]. In response to skeletal muscle injury, deletion of MAPK phosphatase-5 reduces the activation of the mitochondrial apoptotic pathway involving STAT3 and increases the expression of Bcl-2 [122]. A microarray analysis of differentially expressed genes revealed that the IL-6/JAK/STAT3 signaling pathway was strongly activated in denervation-induced skeletal muscle atrophy, while inhibition of the JAK/STAT3 signaling pathway suppressed mitochondrial phagocytosis and muscle atrophy [14]. Furthermore, inhibition of the JAK/STAT3 signaling pathway was found to be sufficient to rescue HCT116 cell-induced myotube atrophy and inhibit the reduction of mitochondrial proteins PGC-1α, OPA1, MFN2, and Cytochrome C in a mouse model of colorectal cancer cachexia [123]. Therefore, therapeutic strategies targeting the JAK-STAT3 pathway may have the potential to delay muscle atrophy.

##### TGF-β-Smad2/3 signaling pathway

TGF-β signals through the activation of classical Smad-dependent and non-classical signaling pathways (e.g. ERK1/2, JNK1/2 and p38 MAPK) and induces muscle atrophy dependent on ROS mechanisms [124]. Targeted inactivation of TGF-β activated kinase 1 (TAK1) can disrupt redox signaling, leading to ROS accumulation, mitochondrial dysfunction and skeletal muscle loss [125, 126]. In a rat model of emphysema, ursolic acid reduces cigarette smoke-induced oxidative stress and muscle atrophy by upregulating IGF-1 and inhibiting the TGF-β1-Smad2/3 signaling pathway [127]. Smad3 deficiency promotes mitochondrial biogenesis and function in white adipose tissue of diabetic and obese mice, as evidenced by increased expression of PGC-1α and increased mitochondrial DNA copy number [128]. Furthermore, in middle-aged adults at high risk of type 2 diabetes, activated TGF-β1 signaling can promote impaired motor responses by downregulating the abundance of PGC-1α, AMPKα2, TFAM, and mitochondrial enzymes [129]. Therefore, the TGF-β-Smad2/3 signaling pathway has the potential to induce several types of muscle atrophy, accompanied by mitochondrial dysfunction.

##### NF-κB signaling pathway

Nuclear factor-κB (NF-κB) is a transcription factor that can be rapidly activated by inflammatory cytokines such as TNF-α and is then involved in a range of biological processes, including inflammation and immune response [61]. TWEAK has been shown to enhance Beclin1 expression in C2C12 myotubes by activating NF-κB transcription factors [130]. MiR-155 in exosomes from M1 polarized macrophages promotes endothelial-to-mesenchymal transition and impair mitochondrial function following traumatic spinal cord injury via activating the NF-κB signaling pathway in vascular endothelial cells [131]. In case of obesity and diabetes, inhibition of the NF-κB signaling pathway ameliorates disturbances in mitochondrial respiratory function/morphology, decreases the expression of key mitochondrial genes such as SDHA, ANT-1, UCP3, and MFN2, and attenuates the increase of ROS and the decrease of insulin sensitivity of myotubes [132]. Furthermore, the extent to which NF-κB inhibition alters mitochondrial function depends on age and muscle specificity, and targeted inhibition of the activity of NF-κB in skeletal muscle at the early stage of life to prevent aging related pathology may be detrimental [133]. Inflammation is an important trigger of muscle atrophy and pro-inflammatory cytokines are important precipitating factors of muscle atrophy. The pro-inflammatory signaling pathway NF-κB can be involved in muscle atrophy due to multiple causes via the regulation of mitochondrial function, which also provides new potential targets for the treatment of muscle atrophy.

#### Mitochondrial factors participate in mitochondrial dysfunction

##### Decreased mitochondrial quality and quantity

Mitochondria in aged skeletal muscle appear larger, rounder in shape, vacuolated in matrix, and shorter in cristae compared with young skeletal muscle mitochondria. Also older muscles lose mitochondria, which may lead to loss of skeletal muscle [134]. Drp1-deficient mitochondria are morphologically large and functionally abnormal, which leads to increased mitochondrial Ca^2+^ uptake, and induces the activation of UPS and unfolded protein reaction (UPR), thereby inducing muscle atrophy [135]. Opa1 deficiency can also change mitochondrial morphology and function and lead to endoplasmic reticulum stress, which then affects muscle mass and metabolic homeostasis [136]. Alterations in mitochondrial morphology, quality and quantity are direct factors causing mitochondrial dysfunction.

##### Mitochondrial DNA (mtDNA) mutation

mtDNA consists of light (L) and heavy (H) strands, carries 16,569 base pairs and contains 37 genes (9 in the L strand and 28 in the H strand), encoding 13 subunits of the electron transfer chain complex I, III, IV and V [137, 138]. Somatic mtDNA deletion mutations expand clonally within individual fibers until a phenotypic threshold is exceeded, leading to the loss of cellular respiration, myofiber atrophy, apoptosis, and necrosis, accompanied by fiber breakage and loss [139]. In muscle fibers, the process begins with an mtDNA replication error that results in 25–80% loss of the mitochondrial genome [140]. In aging skeletal muscle, mtDNA replication damage leads to increased ROS production [141]. The involvement of mtDNA is required for the synthesis of new mitochondria and therefore mtDNA is essential for the maintenance of normal mitochondrial state and biogenesis.

##### Disorder of mitochondrial autophagy

Both excessive autophagy (excessive catabolism) and insufficient autophagy (protein accumulation, oxidative stress, and apoptosis) may lead to muscle atrophy [142]. There is an increase in autophagy in laminin α2 chains deficient muscles, suggesting that excessive autophagy leads to muscle atrophy [143]. Muscle-specific deletion of autophagy-related 7 (Atg7) results in abnormal mitochondrial accumulation, sarcoplasmic reticulum expansion, disorganized sarcomere and formation of abnormal concentric membrane structures, leading to muscle atrophy and impaired muscle function [144]. Furthermore, AMPK activation promotes mitophagy by enhancing mitochondrial fission (via MFF phosphorylation) and autophagosome phagocytosis (via TBK1 activation) in a PINK1-Parkin-independent manner [145]. In X-linked myopathies, mutations in the VMA21 gene reduce lysosomal degradation and result in increased autophagy, leading to autophagic vacuolar myopathy [146, 147]. In a mouse model of diabetic muscular atrophy, there was a 20–30% reduction in muscle mass and myofiber area, characterized by increased microtubule-associated protein light chain 3 (LC3)-containing vesicles and elevated LC3-II levels [148]. NRF2-deficient aged mice showed increased expression of LC3-II, P62, and BNIP3, and excessive autophagy due to AMPK and ROS signaling in skeletal muscle, which may be a potential mechanism for sarcopenia [149]. Under normal physiological conditions, mitophagy is necessary for skeletal muscle growth and development, while under the above pathological conditions mitophagy can cause skeletal muscle atrophy.

##### Disorder of ROS production

Nicotinamide adenine dinucleotide phosphate oxidase and mitochondria are two major sources of ROS production in skeletal muscle. ROS is an important cell signal sensor that controls autophagy through a cascade of different signals localized based on its signaling [150]. It has long been proposed that denervation-induced skeletal muscle atrophy is associated with increased mitochondrial ROS generation [151]. IL-6 deficiency has been found to inhibit mitochondrial ROS production by up-regulating PGC-1α expression in sepsis mice, thus alleviating skeletal muscle atrophy [152]. In addition, aberrant levels of ROS can disrupt the redox environment in older muscles, possibly disrupting cellular signaling and in some cases weakening adaptive responses to exercise [153]. ROS level is closely related to the activation of autophagy, and abnormal ROS production will lead to mitochondrial dysfunction.

##### Disorder of mitochondrial metabolic enzymes

Cytochrome oxidase is the terminal oxidase of the electron transport chain, and citrate synthase activity is closely related to mitochondrial content and is involved in mitochondrial substrate oxidation, which is one of the most commonly used markers of mitochondrial content [154, 155]. With aging, expression of mitophagy protein increases in skeletal muscle, while citrate synthase activity and cytochrome c oxidase subunit IV protein content are considerably reduced [156]. In cancerous cachectic mice, citrate synthase and cytochrome c oxidase activities in skeletal muscle were decreased, damaged mitochondrial content was increased, and Drp1 and MFN2 expression was reduced [157]. Alterations in either mitophagy or mitochondrial dynamics are accompanied by abnormal expression of mitochondrial metabolic enzymes, thereby inducing skeletal muscle atrophy.

##### Destruction of mitochondrial Ca^2+^ homeostasis

Ca^2+^ as ubiquitous signaling second messenger is a key player in the regulation of skeletal muscle cell functions. Ca^2+^ regulates mitochondrial functions, while mitochondria shape Ca^2+^ dynamics [158]. Mitochondrial Ca^2+^ homeostasis plays a pivotal role to maintain muscle homeostasis and to sustain muscle function. The regulation of the mitochondrial Ca^2+^ controls skeletal muscle size, force, and nutrient utilization [159]. Mitochondria capture Ca^2+^ through the mitochondrial calcium uniporter complex, to regulate energy production, cytoplasmic Ca^2+^ signaling, and cell death [160]. The uniporter complex is composed of pore-forming MCU, gatekeeper MICU1 and MICU2, and an auxiliary EMRE subunit essential for Ca^2+^ transport [161]. MICUs sense the changes in cytosolic Ca^2+^ concentrations to switch MCU on and off. When Ca^2+^ levels rise above ~ 1 μM, the uniporter complex is actived [160]. MICUs prevent excessive Ca^2+^ influx that can increase mitochondrial oxidative stress [162]. Reduced MCU activity impedes the autophagic flux, and loss of autophagy further impairs mitochondrial Ca^2+^ signaling, thereby disrupting muscle homeostasis and function [159]. MCU overexpression can prevent denervation-induced skeletal muscle atrophy [163]. Moreover, MCU expression triggers hypertrophy by controlling protein synthesis through the PGC-1α and IGF1-Akt/PKB pathways [163]. Mutations of MICU1 were related to debilitating neuromuscular diseases in patients [164]. In HEK293 cells, removing MICU1 allows mitochondria to more readily take up Ca^2+^. However, the trade-off is elevated ROS, impaired basal metabolism, and higher susceptibility to death [165]. EMRE deletion blocks mitochondrial Ca^2+^ uptake, however, EMRE protein expression is upregulated in a mouse model of muscular dystrophy [166]. Therefore, regulation of mitochondrial Ca^2+^ homeostasis may be a new therapeutic intervention for muscle atrophy.

##### Dysfunction of mitochondrial permeability transition pore (mPTP)

Disruption of mitochondrial membrane potential is the main sign of mitochondrial dysfunction. Mitochondrial membrane potential reduction is associated with an impaired mitochondrial electron transport chain, reduced metabolic oxygen consumption, ATP depletion, and low energy metabolism [167]. The mPTP is a large conductive pore in the inner mitochondrial membrane that is predominantly closed under non-stress conditions. The mPTP is permeable to solutes up to 1.5 kDa in size, and sustained pore opening leads to the dissipation of the mitochondrial membrane potential, organelle swelling, and eventual rupture [168]. The mPTP opening can be induced by increased ROS and Ca^2+^ or mitochondrial depolarization, and it can be further exacerbated by an imbalanced Ca^2+^ homeostasis [169]. Cyclophilin D is involved in the regulation of mPTP. Although loss or inhibition of cyclophilin D confirmed that it was an activator of mPTP opening, with higher levels of Ca^2+^, mPTP opening was cyclophilin D independent [168]. Cyclophilin D was upregulated in skeletal muscle of SMNKO mice (skeletal muscle-specific nicotinamide phosphoribosyl transferase KO) from 2 weeks of age, with associated increased sensitivity of mitochondria to the Ca^2+^-stimulated mPTP opening [170]. Cyclophilin D can bind to adenine nucleotide translocator (ANT), which suggested that the ANT might be the pore-forming unit [168]. Moreover, the expression of pro-apoptotic Bcl-2 family members, Bax and Bak localization to the outer mitochondrial membrane is required for MPTP-dependent mitochondrial dysfunction and subsequent necrotic cell death. Mitochondrial Ca^2+^ retention capacity and MPTP sensitivity are influenced by Bax/Bak activation/oligomerization on the outer mitochondrial membrane [171]. Sporadic denervation in mouse muscle has been shown to reduce mitochondrial respiratory capacity and increase sensitivity to mPTP opening [172]. Furthermore, mitochondrial permeability shift has been identified as a new pathogenesis of skeletal muscle atrophy, which acts through mitochondrial ROS emission and Caspase-3 activation [173]. It is worth noting that the mPTP activation is associated with mitochondrial Ca^2+^ homeostasis and mitochondrial ROS, which together influence mitochondrial function.

##### Decreased mitochondrial cardiolipin

Cardiolipin is the representative phospholipid of the inner mitochondrial membrane and affects the stability of many inner membrane protein complexes, including respiratory chain complexes and metabolite carriers [174]. Cardiolipin that is externalized to the outer mitochondrial membrane may mediate targeted autophagy of mitochondria in primary and transformed neuronal cells by interacting with the autophagy protein LC3 [175]. During long-term muscle inactivity, a decrease in cardiolipin abundance and the relative composition of its fatty acid chains can directly influence mitochondrial function and cause disuse muscle atrophy [23]. Cardiolipin is essential for mitochondrial function and cardiolipin-targeted treatments have significant implications for the prevention of mitochondrial dysfunction.

#### Mitochondrial dysfunction in different disease states

##### Aging and sarcopenia

Sarcopenia is an age-related debilitating skeletal muscle atrophy syndrome. Sarcopenia is one of the main causes that limit daily activities in the elderly, characterized by loss of skeletal muscle mass, decreased muscle strength and decreased physical performance [176]. A hallmark of aging is the loss of protein homeostasis, which is partly due to alterations in the UPS and ALP that lead to impaired function and maintenance of mass in tissues such as skeletal muscle [177]. Sarcopenia patients have reduced expression and activity of mitochondrial respiratory complexes and downregulated oxidative phosphorylation and mitochondrial protein homeostasis genes in skeletal muscle [178]. In an mtDNA mutant mouse model, mtDNA mutations are determinants of mitochondrial electron transport chain function and therefore mitochondrial bioenergetics and ATP homeostasis are impaired, thereby inducing skeletal muscle apoptosis and sarcopenia [179]. During aging, the morphology, quality and quantity of mitochondria are altered, mitochondrial function is also impaired, and skeletal muscle function is lost.

##### Disuse muscle atrophy

Disuse muscle atrophy indicates a temporary condition of unused muscles, such as limb immobilization (i.e., plaster immobilization) due to fracture, bed rest, hindlimb unloading, denervation, and heart failure. Disuse muscle atrophy promotes significant mitochondrial alterations, leading to impaired metabolic homeostasis and an increased degree of muscle atrophy [180]. However, a study indicated that during the progression of disuse muscle atrophy, mitochondrial aberrations exert different effects on male and female mice, and females may give up muscle mass to maintain mitochondrial mass compared with males, which may lead to different clinical manifestations of atrophy [181]. Chronic muscle inactivity can lead to major disturbances in intracellular calcium homeostasis, resulting in impaired mitochondrial calcium handling and increased oxidant production [182]. It has been demonstrated that denervation induces a reduction in mitochondrial biogenesis in response to changes in mitochondrial translation factors in mouse skeletal muscle, which provides new molecular-level insights into the effects of muscle denervation on mitochondrial translation processes [183]. In addition, miR-142a-5p is an important regulator of denervation-induced skeletal muscle atrophy, which can induce mitochondrial dysfunction, mitophagy and apoptosis by targeting MFN1 [184]. Spinal cord injury is a unique form of disuse atrophy in which paralysis and disruption of the central nervous system leads to a rapid decline in skeletal muscle function and metabolic status, as well as disruption of the activity of PGC-1α and calcium-regulated neurophosphatase that are key regulators of mitochondrial health and function [185]. Obvious mitochondrial dysfunction is a very important mechanism in the process of disuse muscle atrophy.

##### Cachexia

The progression of cachexia is associated with metabolic changes, mainly including excessive energy expenditure, increased proteolysis and mitochondrial dysfunction [186]. Muscle atrophy associated with mitochondrial dysfunction can be observed in cachectic rodents, including impaired mitochondrial dynamics (increased fission (Fis1) and reduced fusion (MFN1 and MFN2)), reduced activity of respiratory chain complexes, and an increase in the suspected indicators of mitochondrial energy coupling (UCP-2 and UCP-3) [187]. The ALP and UPS are simultaneously activated in skeletal muscle of patients with cachectic gastric cancer and may play a coordinated role in cachexia-induced muscle loss [188]. A genome-wide transcriptome analysis of a cancer-induced cachexia rodent model indicated that the expression of genes involved in mitochondrial fusion and fission, ATP production and mitochondrial density was reduced, while the expression of genes involved in ROS detoxification and mitochondrial phagocytosis was increased [189]. In addition, autophagy exacerbated muscle atrophy and impaired mitochondrial function in a C26 mouse model of cancer cachexia [190]. Therefore, mitochondrial dynamics, mitophagy and oxidative stress disorder are the main causal factors for cachexia.

##### Chronic kidney disease (CKD)

CKD has multiple causative factors, commonly characterized by a recurrent cycle of glomerular or tubular epithelial injury, and elevated intracellular ROS levels play a major role in the pathogenesis of CKD [191]. CKD predisposes to acute kidney injury (AKI), while AKI facilitates CKD progression [192]. Dysregulation of mitochondrial homeostasis, bioenergetics alterations and organelle stress crosstalk also contribute to the transition from AKI to CKD [193]. Following CKD, there is a reduction in mitochondrial copy number, expression of biogenesis markers (PGC-1α and TFAM) and mitochondrial fusion marker (Mfn2) while an increase in BNIP3, Beclin-1 and LC3II at protein and mRNA levels, indicating the formation of autophagosome [194]. Both mitochondrial dysfunction and reduced antioxidant enzyme levels can increase ROS production, which often cause oxidative stress and lead to CKD-induced muscle atrophy [4, 195]. In addition, FoxO3 can activate autophagy in skeletal muscle of CKD patients, which may be a novel intervention for muscle atrophy in CKD [196]. Mitochondrial bioenergetics plays a central role in CKD, and targeting metabolic pathways involved in mitochondrial bioenergetics is a promising therapeutic strategy.

##### Diabetes

Diabetes can reduce skeletal muscle function, leading to myasthenia and muscle atrophy and causing structural changes such as metabolic disturbances characterized by reduced cellular glucose uptake and fatty acid oxidation, impaired mitochondrial function, and muscle fiber conversion [6]. Oxidative stress can induce insulin deficiency and produce large amounts of ROS to block insulin signaling, thus triggering insulin resistance, which may help the malignant progression of diabetic muscular atrophy [6]. In addition, in diabetic mice, mitochondrial number and quality were decreased, and mitophagy and biogenesis-related proteins were significantly reduced [197]. However, there are not many drugs targeting mitochondria for treatment of diabetic muscular atrophy. So, the search for biological targets based on the molecular mechanisms of diabetic muscular atrophy is required for the purpose of developing new drugs.

##### Obesity

Obesity causes structural and functional changes in skeletal muscle, leading to the accumulation of intramuscular lipids, which is associated with impaired mitochondrial content and function in skeletal muscle [198]. Obesity also leads to renal mitochondrial dysfunction and energy imbalance, accelerating the progression of CKD and worsening CKD-dependent sarcopenia in mice [199]. Compared with sarcopenia or obesity alone, myopenic obesity is more likely to increase the risk of death, and patients with myopenic obesity have significantly lower expression of mitochondria-related proteins PGC-1α, MFN1, MFN2 and DRP1 than normal controls [200]. Furthermore, mitochondrial uncoupling may provide protection against myopenic obesity via enhancing skeletal muscle mitophagy and quality control to attenuate age-related decline in muscle mass and function [201]. Targeted regulation of mitochondrial dynamics, mitochondrial biogenesis and mitophagy seems to be an attractive treatment strategy for muscle atrophy caused by Obesity.

##### Amyotrophic lateral sclerosis (ALS)

ALS is a neurodegenerative disease accompanied by progressive loss of motor neurons, eventually leading to fatal paralysis [202, 203]. ALS was initially thought to be associated with oxidative stress, as it was first shown to be associated with the mutant SOD1, TDP-43 or other ALS-related mutant proteins that can all lead to mitochondrial imbalance in ALS and affect mitochondrial respiration as well as ATP production, calcium handling, mitochondrial dynamics and apoptotic signaling [204, 205]. ROS can lead to mitochondrial DNA mutations, membrane permeability and calcium homeostasis, as well as enhanced lipid oxidation and protein carbonylation, which will lead to various neurodegenerative diseases, including ALS [206]. Furthermore, a serum lipid analysis revealed a significant decrease in Cardiolipin content in the spinal cord of ALS rats, along with the loss of mitochondrial integrity [207]. Mitochondria are directly involved in the pathogenesis of ALS, but the causal relationship between mitochondrial dysfunction and ALS pathogenesis remains to be confirmed.

##### Spinal muscular atrophy (SMA)

SMA is caused by loss of function of survival motor neuron (SMN) protein, resulting in structural and functional alterations of the cytoskeleton in motor neurons and other cells [208]. SMN has been shown to affect mitochondrial and bioenergetic pathways and regulate the UPS function [209]. Impaired mitochondrial biogenesis can be observed both in the muscles o SMA patients and in the motor neurons of SMA mice, while the expression of the mitochondria-related genes TFAM, NRF1 and NRF2 is downregulated in the muscles of SMA patients [210]. In addition, SMA can increase the levels of oxidative stress and impair mitochondrial membrane potential in motor neurons, and fragmentation of mitochondrial networks in primary motor neurons of SMA mice is significantly increased [211]. The pathogenesis of SMA involves the proteolysis system and mitochondrial dysfunction, and their effects on SMN are required for further exploration.

##### Spinal and bulbar muscular atrophy (SBMA)

SBMA is an inherited neuromuscular disease characterized by motor neuron deficiency and skeletal muscle atrophy caused by polyglutamine expansion in the androgen receptor gene. Altered autophagy and mitochondrial defects underlie SBMA neuromuscular degeneration [212, 213]. In impaired motor neurons, an elevated synaptojanin 2 binding protein (SYNJ2BP) level (an outer mitochondrial membrane protein) alters the cellular distribution of mitochondria and increases mitochondrial-endoplasmic reticulum membrane contact sites, while lowering the SYNJ2BP level improves mitochondrial oxidative function [214]. Molecular links between epigenetic dysregulation of SBMA motor neurons and mitochondrial damage and metabolic dysfunction have been identified using gene expression analysis and ChIP sequencing [215]. These findings highlight the impact of mitochondrial dysfunction on SBMA and the search for potential biological targets is an urgent task for us.

##### Duchenne muscular dystrophy (DMD)

DMD is one of the most common and severe forms of muscle atrophy caused by mutations in the DMD gene encoding different isoforms of antimyotrophic proteins [216, 217]. DMD protein deficiency leads to intracellular Ca^2+^ dysregulation, mitochondrial dysfunction and increased ROS production [218]. Mitochondrial dysfunction is one of the first cellular changes in myofibers following DMD, as evidenced by mitochondrial dysfunction, abnormal mitochondrial morphology and mitophagy impairment (degradation of damaged mitochondria) [219]. In an mdx mouse model at 10–12 weeks of age, functional mitochondrial oxidative capacity was found to be disturbed, suggesting that mild oxidative stress reduces oxidative phosphorylation and thus declines ATP production [220]. In the future, we should pay attention to the impact of mitochondria-related pathways on DMD, which will lead to further understanding of the molecular mechanisms of DMD and potentially facilitate the discovery of DMD-targeted mitochondrial therapies.

## Therapeutic strategies targeting mitochondria for skeletal muscle atrophy

**Fig. 4:**
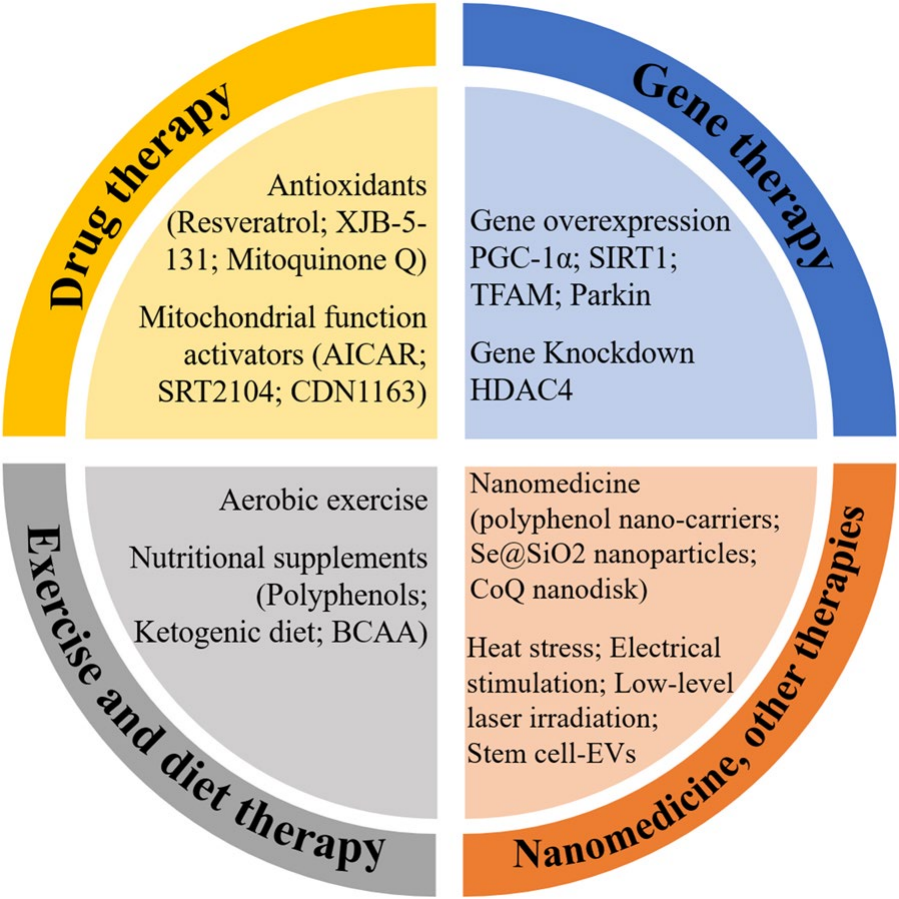
Therapeutic strategies targeting mitochondria for muscle atrophy. Strategies mainly include drug therapy, exercise and diet therapy, gene therapy and other therapies

Mitochondria can provide sufficient energy for the life activities in cells. Mitochondrial dysfunction has been shown to play a very important role in the process of skeletal muscle atrophy. Targeted mitochondrial therapy has become an effective strategy, can directly regulate mitochondria, and improve treatment efficiency in skeletal muscle atrophy. At the same time, direct targeting of mitochondria may lead to fewer side effects on normal tissues. Targeted mitochondrial therapy has good biomedical prospects and is expected to provide new directions for clinical diagnosis and treatment. Strategies for improving mitochondrial function and delaying muscle atrophy mainly include mitochondria-targeted drug therapy (Mitochondria-targeted antioxidants, mitochondrial function activators, etc.), exercise and diet therapy, mitochondria-targeted gene therapy and other therapies (Fig. 4).

### Mitochondria-targeted drug therapy

#### Mitochondria-targeted antioxidants

So far, more and more mitochondrial targeting drugs have been reported. Meanwhile, the effective way to deliver drugs specifically to mitochondria is by covalent linking a lipophilic cation such as an alkyltriphenylphosphonium moiety to a pharmacophore of interest [221]. The combination of metformin and exercise improves mitochondrial bioenergetics and has beneficial effects against muscle loss and fat accumulation by regulating redox status [222]. Moreover, Mito-Met (metformin conjugated with TPP +) can enhance the targeting of metformin [221]. Resveratrol prevents high-fat diet-induced muscle atrophy in aged rats by reversing mitochondrial dysfunction and oxidative stress via the PKA/LKB1/AMPK pathway [223]. The mitochondria-targeted antioxidant XJB-5-131 increases the activity of electron transfer chain complexes in skeletal muscle mitochondria, reversing age-related alterations in mitochondrial function and improving contractility of skeletal muscles [224]. Mitoquinone Q improves mitochondrial homeostasis and metabolism, promotes β-oxidation in muscle tissue, and facilitates the glycolytic-oxidative transition in muscle metabolism and fiber composition [225]. Mitochondrial cardiolipid-targeted peptide, SS peptide, restores mitochondrial function, remodels mitochondria, repairs cellular structure, and promotes tissue regeneration during aging [226]. Szeto-Schiller 31, a mitochondria-targeted antioxidant peptide, prevents inactivity-induced decreased mitochondrial coupling and increased ROS emission to protect mitochondrial function and prevent muscle atrophy due to prolonged inactivity [23]. Szeto-Schiller 31 has shown promise in restoring mitochondrial bioenergetics viability in a phase I-II trial targeting heart failure and primary mitochondrial myopathy [227]. Astaxanthin, a red lutein carotenoid, promotes muscle health by reducing oxidative stress, myogenic apoptosis, and proteolysis pathways, and promoting mitochondrial regeneration and angiogenesis [228]. Trimetazidine, a partial inhibitor of lipid oxidation, prevents high-fat diet-induced muscle dysfunction by improving mitochondrial quality-control and mitochondrial function [229]. In addition, edaravone reverses oxidative stress-induced apoptosis and inhibits upregulation of mitochondrial ROS in induced pluripotent stem cell-derived spinal motor neurons from SMA patients. Therefore, it may be a therapeutic target for SMA [230]. Dysregulation of oxidative stress levels has been shown to be involved in the progression of skeletal muscle atrophy, so the specific use of antioxidants to target and modulate mitochondrial function would be a very effective intervention for muscle atrophy.

#### Mitochondrial function activators

5-Aminoimidazole-4-carboxamide ribonucleoside (AICAR) is an activator of AMPK that effectively improves mitochondrial and muscle function while maintaining the size of skeletal muscle in models of sarcopenia and cancer cachexia [2]. The age-related decreases in NRF2 signaling activity and mitochondrial dysfunction may be associated with the development of age-related diseases. Sulforaphane is a natural NRF2 activator. Cohorts of 2 month-old and 21- to 22 month-old mice were administered regular rodent diet or diet supplemented with Sulforaphane for 12 weeks. Sulforaphane restored Nrf2 activity, mitochondrial function, exercise capacity, glucose tolerance, and activation/differentiation of skeletal muscle satellite cells [231]. Furthermore, sulforaphane improves muscle function and pathology and protects dystrophic muscle from oxidative damage related to the NRF2 signaling pathway in mdx mice, with clinical implications for the treatment of patients with sarcopenia [232]. SRT2104 is a synthetic small molecule activator of SIRT1, and SRT2104 treatment improves systemic metabolic function, increases mitochondrial content, and preserves bone and muscle mass in an experimental model of atrophy [233]. CDN1163 is a novel small molecule allosteric activator of sarcoplasmic reticulum Ca^2+^ ATPase (SERCA) that reverses increased mitochondrial ROS generation and increased oxidative damage in muscle tissue in SOD1 mice, preventing oxidative stress-related muscle atrophy and weakness [234]. These activators can directly target molecules involved in mitochondrial dysfunction, providing potential targets for the development of new drugs to prevent and treat skeletal muscle atrophy.

#### Other drugs

During skeletal muscle aging, *Lactobacillus paracasei* PS23 protects mitochondrial function by reducing age-associated inflammation and ROS emission, thereby slowing age-related muscle loss [235]. In a mouse model of disuse muscle atrophy, Gomisin G (a lignan component of S. chinensis) enhances mitochondrial biogenesis and function through the SIRT1/PGC-1α signaling pathway to improve muscle strength [236]. Ginsenoside Rg3 protects against glucocorticoid-induced muscle atrophy by improving mitochondrial biogenesis and myotubular growth through the activation of PGC-1α [237]. Geranylgeraniol attenuates muscle atrophy in the flounder muscle of diabetic rats by altering mitochondrial mass [238]. Myricanol prevents against dexamethasone-induced muscle atrophy and myasthenia, reduces muscle protein degradation, enhances autophagy, and promotes mitochondrial biogenesis and function by activating SIRT1 in mice [239]. Dihydromyricetin reverses mitochondrial dysfunction via PGC-1α/TFAM and PGC-1α/MFN2 signaling pathways to attenuate dexamethasone-induced muscle atrophy [240]. Chrysanthemi Zawadskii var. Latilobum attenuates mitochondrial dysfunction in skeletal muscle of obese mice by modulating protein arginine methyltransferases, thereby alleviating obesity-induced skeletal muscle atrophy [241]. Paeoniflorin increases the activity of electron transport chain complexes and mitochondrial membrane potential, and improves skeletal muscle atrophy in CKD through AMPK/SIRT1/PGC-1α-mediated oxidative stress and mitochondrial dysfunction [242]. Urolithin A alleviates the symptoms of DMD by inducing mitophagy, increases skeletal muscle respiratory capacity, and improves muscle regeneration, which may have potential therapeutic applications in sarcopenia [243]. Olesoxime, a mitochondria-targeted drug for SMA, has been in phase III clinical trials. Olesoxime exerts its neuroprotective effects through modulation of mPTP to improve cell survival in multiple in vitro and in vivo models [244]. Oyster hydrolysate is a valuable natural material to inhibit skeletal muscle atrophy by regulating protein turnover and mitochondrial biogenesis [245]. Celecoxib alleviates denervation-induced muscle atrophy by reducing mitophagy and inhibiting oxidative stress [5]. Aspirin alleviates denervation-induced muscle atrophy and inhibits the shift from type I to type II muscle fiber and mitophagy via the SIRT1/PGC-1α axis and STAT3 signaling [246]. Levetiracetam is neuroprotective against SMA by ameliorating mitochondrial dysfunction in spinal motor neurons differentiated from SMA patient-derived induced pluripotent stem cells [247]. Mitochondria-targeted interventions using L-carnitine or teneligliptin can be used to treat CKD-induced muscle atrophy and decreased exercise tolerance [248]. Furthermore, ATG-125 is a phytochemical-rich herbal formulation that hinders sucrose-induced gastrocnemius muscle atrophy via rescuing Akt signaling and improving mitochondrial dysfunction in young adult mice [249]. Treatments with the pro-appetitive hormone ghrelin significantly increase mitochondrial respiratory capacity of C2C12 cells enhance muscle anabolism, and play an important role in preserving aging muscle [250]. In addition to antioxidants and activators, many other drugs such as Chinese herbs, anti-inflammatory drugs, and hormones can alleviate skeletal muscle atrophy by improving mitochondrial function. So, we need to focus on their long-term efficacy and safety.

### Nanomedicine

In recent years, nanomedicine technology targeting mitochondria or cells has attracted increasing attention. Compared to conventional approaches, drug targeting with nanomaterials improves biocompatibility, safety, and specificity [251]. For example, nanomaterials have been used to enhance and mediate the functions of vascular cells (such as vascular endothelium and smooth muscle cells) and to prevent thrombosis and inflammation on stents in cardiovascular disease [251]. The polyphenols show protective antioxidant role in neurodegenerative disease at least partially due to their capacity to stimulate mitochondrial biogenesis and improve their function, which elevates mitochondrial efficiency resulting in diminished ROS production [252]. However, polyphenol compounds possess weak pharmacokinetics properties such as low bioavailability and solubility. The targeted polyphenol delivery by drug carriers created using nanotechnology that guarantees target specificity can overcome the drawbacks, and boost the bioavailability and stability of the therapeutic molecules in vivo [251]. Moreover, researches show that porous Se@SiO2 nanoparticles which would slowly release selenium could improve oxidative injury to promote muscle regeneration via modulating mitochondria [253]. CoQ’s lack of aqueous solubility and poor oral bioavailability contribute to suboptimal results observed with respect to the effect of CoQ supplements on statin-induced myopathy. Importantly, the nanodisk enhances CoQ bioavailability that represent a water-soluble vehicle capable of delivering CoQ to cultured myotubes [254]. However, there are still many challenges, such as the fabrication ad toxicity of nanomaterials. Such as, copper nanoclusters are increasingly being used in nanomedicine owing to their utility in cellular imaging and as catalysts. Additionally, exposure to CuNCs may be a risk factor for the skeletal muscle system [255]. More thorough studies of nanomedicine are still needed.

### Exercise and diet

Exercise triggers an increase in key regulatory components of mitochondrial biogenesis (e.g., PGC-1, NRF1, and NRF2), and PGC-1 mediates a coordinated increase in GLUT4 and mitochondria [256]. Moreover, exercise attenuates UPS activity and increases the expression of mitophagy-related genes in skeletal muscle of patients with inflammatory myopathies [257]. It has been reported that exercise may inhibit muscle apoptosis, stimulate mitochondrial oxidative capacity and increase muscle blood flow by activating mTOR signaling and decreasing local TNF-α levels, thereby reversing sarcopenia in patients with cirrhosis [258]. In addition, aerobic exercise may help to inhibit the loss of mitochondrial content in skeletal muscle and forestall aging-induced complications of skeletal muscle, such as sarcopenia and insulin resistance [259]. Aerobic exercise can not only increase SIRT3 and PGC-1α expression levels in sedentary, overweight or obese adolescents, but it also enhances amino acid and carbohydrate intake in healthy older adults, which may prevent against muscle loss with age [260, 261]. Aerobic exercise has also been reported to improve mitochondrial function via Sestrin2 in an AMPKα2-dependent manner in sarcopenia mice [262]. Sedentary individuals present with decreased expression of skeletal muscle catabolism-related proteins (e.g., FoxO3a and MSTN), improved mitochondrial dynamics, and significant activation of signaling pathways associated with proliferation after aerobic exercise training, thereby favoring an increase in muscle fiber and overall muscle size, which may be associated with skeletal muscle hypertrophy [263]. Although endurance exercise training has long been thought to elevate aerobic capacity of skeletal muscle by enhancing mitochondrial quality-control and mitochondrial function, alternative exercise training that can induce similar improvements in mitochondria is gaining increasing attention as a viable intervention [264]. However frequent unaccustomed exercise can alter the structure and function of skeletal muscle fibers, which is called exercise-induced muscle damage. Exercise-induced muscle damage can lead to a temporary muscle damage and soreness that negatively affects muscle function [265]. In addition to exercise, attention to diet can also have a therapeutic effect on muscle atrophy. As nutritional supplements, polyphenols are plant-based compounds with antioxidant and anti-inflammatory properties, many of which are beneficial to human health and may delay skeletal muscle loss and functional impairment [266]. Long-term ketogenic diet slows aging-related muscle mass loss and increases mitochondrial content in aging skeletal muscle [267]. A ketogenic diet enhances mitochondrial biogenesis, oxidative metabolism, and antioxidant capacity in mice, and may protect skeletal muscle mass and function in aged mice [268]. Ketogenic diets combined with exercise alter mitochondrial function in human skeletal muscle while improving metabolic health [269]. In addition, branched-chain amino acids (BCAA: leucine, valine, isoleucine) have been shown to maintain body mass and cardiac function and prolong survival in rats with heart failure, possibly by increasing the expression of genes involved in mitochondrial biogenesis and skeletal muscle function [270]. Leucine is a branched-chain amino acid supplement that activates mTORC1, promotes protein synthesis and inhibits autophagy in muscle [271, 272]. Whereas drug therapy may carry risks such as side effects, exercise and diet seem to be relatively healthy treatments that will benefit the body from all aspects if adhered to over time.

### Mitochondria-targeted gene therapy

Mitochondria belong to semi-automatic organelles, which have their own genome different from nuclear genome. Targeting genes involved in mitochondrial regulation and mitochondrial genes are of great significance for the treatment of skeletal muscle atrophy. PGC-1α overexpression preserves muscle size by inhibiting ALP and UPS and alleviating mitochondrial dysfunction, indicating that compounds that induce PGC-1α expression may benefit the treatment of muscle atrophy [273]. Overexpression of TFAM reduces skeletal muscle atrophy after hindlimb suspension in mice, which is correlated with the increased expression of antioxidants [274]. Parkin overexpression may prevent sepsis-induced skeletal muscle atrophy by improving mitochondrial mass and content [275]. Elevated SIRT1 expression leads to an increase in oxidative metabolism and mitochondrial biogenesis markers, thereby improving pathophysiological manifestations in a mouse model of DMD [276]. Histone deacetylase 4 (HDAC4) may inhibit mitophagy in denervated skeletal muscle and improve mitochondrial function through the direct regulation of myogenin, and therefore, the histone deacetylase 4-myogenin axis may function as a new target for the prevention and treatment of muscle atrophy [9]. Inhibition of the IL-6/JAK/STAT3 signaling pathway inhibits muscle atrophy and mitophagy, accompanied by a reduction in the expression of atrophy-related and autophagy-related genes, so the IL-6/JAK/STAT3 pathway can be used as a targeted strategy for skeletal muscle atrophy [14]. Furthermore, restoration of miR-181a levels in aged mice can prevent the accumulation of p62, DJ-1 and PARK2 and improve mitochondrial mass and muscle function [277]. lncRNA EDCH1 may improve mitochondrial function through SERCA2-mediated activation of the AMPK pathway to diminish muscle atrophy [278]. Early activation of lncRNA Pvt1 following muscle atrophy affects mitochondrial respiration and morphology and influences autophagy and apoptosis related to mitochondrial conformation and myofiber size, and thus targeting lncRNA Pvt1 may be a viable therapeutic target for muscle atrophy [279]. lncRNA Gm20743 may be involved in regulating mitochondrial function, oxidative stress, cell proliferation and myotube differentiation in skeletal muscle cells, and may be a potential therapeutic target for diabetes-induced sarcopenia [280]. Taken together, these genes and non-coding RNAs would be novel targets for targeting mitochondrial dysfunction in the treatment of muscle atrophy.

### Other treatments

Extracellular vesicles are thought to be involved in many physiological and pathological processes, such as cancer progression, immune regulation, neurodegenerative diseases, and tissue regeneration [13]. Extracellular vesicles derived from skin precursor-derived Schwann cells can reduce mitochondrial vacuolar degeneration and autophagy in denervated muscles by inhibiting autophagy-associated proteins and alleviate muscle atrophy by suppressing oxidative stress and inflammatory responses [50]. Human umbilical cord-derived mesenchymal stromal cells ameliorate sarcopenia-associated skeletal muscle atrophy and dysfunction through AMPK-PGC-1α axis-mediated anti-apoptotic, anti-inflammatory, and mitochondrial biogenesis mechanisms [281]. Mesenchymal stem cell can mediate the transplantation of mitochondria into aging cells. This can restore the function of mitochondria in aging muscle cells and neurons, and then achieve the therapeutic purpose [282]. In addition, heat stress has been shown to trigger a stress response that leads to increased heat shock protein expression and improved mitochondrial function, while attenuating the reduction in human skeletal muscle mass and metabolic function due to immobilization [283]. Electrical stimulation can prevent against doxorubicin-induced muscle atrophy and mitochondrial loss in C2C12 myotubes [284]. Low-level laser irradiation prevents doxorubicin-induced skeletal muscle atrophy by preserving mitochondrial homeostasis and alleviating oxidative stress and apoptosis through the AMPK/SIRT1/PCG-1α pathway [285]. It has also been proposed that caloric restriction can delay sarcopenia by reducing oxygen radical production, decreasing oxidative stress damage, enhancing mitochondrial function, improving protein homeostasis, reducing iron overload, increasing autophagy and apoptosis, and reducing inflammation [286]. In conclusion, there are many ways to target mitochondria for the treatment of sarcopenia, and their mechanism is more worthy of our attention. In the future, the combined therapeutic modalities may be an alternative.

## Prospects

Our understanding of the role of mitochondria in skeletal muscle atrophy has progressed considerably over the last few years. Mitochondria play a very important role in skeletal muscle growth and development, and mitochondrial dysfunction is an important cause of skeletal muscle atrophy. Therefore, the molecular mechanisms by which mitochondrial dysfunction induces skeletal muscle atrophy have attracted the interest of scientists. An understanding of these mechanisms could benefit the development of clinical treatment options for skeletal muscle atrophy, and future therapeutic strategies targeting mitochondria may be a key measure to prevent or treat different types of skeletal muscle atrophy. Currently the common therapeutic approaches are drug therapy, gene therapy, stem cell therapy and physiotherapy, and if additional combination therapeutic strategies can be developed or the feasibility of mitochondrial transplantation can be increased, the quality of life would be greatly improved in patients with muscle atrophy.

## Data Availability

Not applicable.

## References

[1] Ji Y, Li M, Chang M, Liu R, Qiu J, Wang K, Deng C, Shen Y, Zhu J, Wang W (2022). Inflammation: roles in skeletal muscle atrophy. Antioxidants.

[2] Yan Y, Li M, Lin J, Ji Y, Wang K, Yan D, Shen Y, Wang W, Huang Z, Jiang H (2022). Adenosine monophosphate activated protein kinase contributes to skeletal muscle health through the control of mitochondrial function. Front Pharmacol.

[3] Guo M, Yao J, Li J, Zhang J, Wang D, Zuo H, Zhang Y, Xu B, Zhong Y, Shen F (2023). Irisin ameliorates age-associated sarcopenia and metabolic dysfunction. J Cachexia Sarcopenia Muscle.

[4] Wang K, Liu Q, Tang M, Qi G, Qiu C, Huang Y, Yu W, Wang W, Sun H, Ni X (2022). Chronic kidney disease-induced muscle atrophy: molecular mechanisms and promising therapies. Biochem Pharmacol.

[5] Zhang L, Li M, Wang W, Yu W, Liu H, Wang K, Chang M, Deng C, Ji Y, Shen Y (2022). Celecoxib alleviates denervation-induced muscle atrophy by suppressing inflammation and oxidative stress and improving microcirculation. Biochem Pharmacol.

[6] Shen Y, Li M, Wang K, Qi G, Liu H, Wang W, Ji Y, Chang M, Deng C, Xu F (2022). Diabetic muscular atrophy: molecular mechanisms and promising therapies. Front Endocrinol.

[7] Wang W, Li M, Chen Z, Xu L, Chang M, Wang K, Deng C, Gu Y, Zhou S, Shen Y (2022). Biogenesis and function of extracellular vesicles in pathophysiological processes of skeletal muscle atrophy. Biochem Pharmacol.

[8] Huang L, Li M, Deng C, Qiu J, Wang K, Chang M, Zhou S, Gu Y, Shen Y, Wang W (2023). Potential therapeutic strategies for skeletal muscle atrophy. Antioxidants.

[9] Sartori R, Romanello V, Sandri M (2021). Mechanisms of muscle atrophy and hypertrophy: implications in health and disease. Nat Commun.

[10] Vainshtein A, Sandri M (2020). Signaling pathways that control muscle mass. Int J Mol Sci.

[11] Shen Y, Zhang R, Xu L, Wan Q, Zhu J, Gu J, Huang Z, Ma W, Shen M, Ding F, Sun H (2019). Microarray analysis of gene expression provides new insights into denervation-induced skeletal muscle atrophy. Front Physiol.

[12] Huang Z, Fang Q, Ma W, Zhang Q, Qiu J, Gu X, Yang H, Sun H (2019). Skeletal muscle atrophy was alleviated by salidroside through suppressing oxidative stress and inflammation during denervation. Front Pharmacol.

[13] Huang Z, Zhu J, Sun J, Ma W, Wang L, Zhang Q, Sun H (2018). Effect of mammalian target of rapamycin signaling pathway on nerve regeneration. Biotarget.

[14] Huang Z, Zhong L, Zhu J, Xu H, Ma W, Zhang L, Shen Y, Law BY, Ding F, Gu X, Sun H (2020). Inhibition of IL-6/JAK/STAT3 pathway rescues denervation-induced skeletal muscle atrophy. Ann Transl Med.

[15] Annesley SJ, Fisher PR (2019). Mitochondria in health and disease. Cells.

[16] Christian BE (2017). Mitochondrial ribosome-binding factor A (mtRBFA): mediator of rRNA methylation and maturation. Biotarget.

[17] Sakellariou GK, Pearson T, Lightfoot AP, Nye GA, Wells N, Giakoumaki II, Vasilaki A, Griffiths RD, Jackson MJ, McArdle A (2016). Mitochondrial ROS regulate oxidative damage and mitophagy but not age-related muscle fiber atrophy. Sci Rep.

[18] Romanello V, Sandri M (2021). The connection between the dynamic remodeling of the mitochondrial network and the regulation of muscle mass. Cell Mol Life Sci.

[19] Carafoli E, Margreth A, Buffa P (1964). Early biochemical changes in mitochondria from denervated muscle and their relation to the onset of atrophy. Exp Mol Pathol.

[20] Matsumoto C, Sekine H, Nahata M, Mogami S, Ohbuchi K, Fujitsuka N, Takeda H (2022). Role of mitochondrial dysfunction in the pathogenesis of cisplatin-induced myotube atrophy. Biol Pharm Bull.

[21] Memme JM, Oliveira AN, Hood DA (2022). p53 regulates skeletal muscle mitophagy and mitochondrial quality control following denervation-induced muscle disuse. J Biol Chem.

[22] Andreux PA, van Diemen MPJ, Heezen MR, Auwerx J, Rinsch C, Groeneveld GJ, Singh A (2019). Publisher correction: Mitochondrial function is impaired in the skeletal muscle of pre-frail elderly. Sci Rep.

[23] Hyatt H, Deminice R, Yoshihara T, Powers SK (2019). Mitochondrial dysfunction induces muscle atrophy during prolonged inactivity: a review of the causes and effects. Arch Biochem Biophys.

[24] Huang L, Li M, Deng C, Qiu J, Wang K, Chang M, Zhou S, Gu Y, Shen Y, Wang W (2022). Potential therapeutic strategies for skeletal muscle atrophy. Antioxidants.

[25] Yadav A, Dahuja A, Dabur R (2021). Dynamics of toll-like receptors signaling in skeletal muscle atrophy. Curr Med Chem.

[26] Ma W, Xu T, Wang Y, Wu C, Wang L, Yang X, Sun H (2018). The role of inflammatory factors in skeletal muscle injury. Biotarget.

[27] Zhang H, Qi G, Wang K, Yang J, Shen Y, Yang X, Chen X, Yao X, Gu X, Qi L (2023). Oxidative stress: roles in skeletal muscle atrophy. Biochem Pharmacol.

[28] Londhe P, Guttridge DC (2015). Inflammation induced loss of skeletal muscle. Bone.

[29] Aoi W, Sakuma K (2011). Oxidative stress and skeletal muscle dysfunction with aging. Curr Aging Sci.

[30] Powers SK, Smuder AJ, Judge AR (2012). Oxidative stress and disuse muscle atrophy: cause or consequence?. Curr Opin Clin Nutr Metab Care.

[31] Musaro A, Fulle S, Fano G (2010). Oxidative stress and muscle homeostasis. Curr Opin Clin Nutr Metab Care.

[32] Chen Z, Chen X, Ji Y, Zhang L, Wang W, Shen Y, Sun H (2021). A narrative review of the role of m6A in oxidative stress and inflammation. Biotarget.

[33] Ma W, Cai Y, Shen Y, Chen X, Zhang L, Ji Y, Chen Z, Zhu J, Yang X, Sun H (2021). HDAC4 knockdown alleviates denervation-induced muscle atrophy by inhibiting myogenin-dependent atrogene activation. Front Cell Neurosci.

[34] Fang WY, Tseng YT, Lee TY, Fu YC, Chang WH, Lo WW, Lin CL, Lo YC (2021). Triptolide prevents LPS-induced skeletal muscle atrophy via inhibiting NF-kappaB/TNF-alpha and regulating protein synthesis/degradation pathway. Br J Pharmacol.

[35] Pascual-Fernandez J, Fernandez-Montero A, Cordova-Martinez A, Pastor D, Martinez-Rodriguez A, Roche E (2020). Sarcopenia: molecular pathways and potential targets for intervention. Int J Mol Sci.

[36] Jackson MJ (1985). Redox regulation of muscle adaptations to contractile activity and aging. J Appl Physiol.

[37] Kim DS, Cha HN, Jo HJ, Song IH, Baek SH, Dan JM, Kim YW, Kim JY, Lee IK, Seo JS, Park SY (2015). TLR2 deficiency attenuates skeletal muscle atrophy in mice. Biochem Biophys Res Commun.

[38] Sharma B, Dutt V, Kaur N, Mittal A, Dabur R (2020). Tinospora cordifolia protects from skeletal muscle atrophy by alleviating oxidative stress and inflammation induced by sciatic denervation. J Ethnopharmacol.

[39] Wu C, Tang L, Ni X, Xu T, Fang Q, Xu L, Ma W, Yang X, Sun H (2019). Salidroside attenuates denervation-induced skeletal muscle atrophy through negative regulation of pro-inflammatory cytokine. Front Physiol.

[40] Mukund K, Subramaniam S (2020). Skeletal muscle: a review of molecular structure and function, in health and disease. Wiley Interdiscip Rev Syst Biol Med.

[41] Sun H, Gong Y, Qiu J, Chen Y, Ding F, Zhao Q (2014). TRAF6 inhibition rescues dexamethasone-induced muscle atrophy. Int J Mol Sci.

[42] He Q, Qiu J, Dai M, Fang Q, Sun X, Gong Y, Ding F, Sun H (2016). MicroRNA-351 inhibits denervation-induced muscle atrophy by targeting TRAF6. Exp Ther Med.

[43] Shen Y, Zhang Q, Huang Z, Zhu J, Qiu J, Ma W, Yang X, Ding F, Sun H (2020). Isoquercitrin delays denervated soleus muscle atrophy by inhibiting oxidative stress and inflammation. Front Physiol.

[44] Khalil R (2018). Ubiquitin-proteasome pathway and muscle atrophy. Adv Exp Med Biol.

[45] Kitajima Y, Yoshioka K, Suzuki N (2020). The ubiquitin-proteasome system in regulation of the skeletal muscle homeostasis and atrophy: from basic science to disorders. J Physiol Sci.

[46] Bodine SC, Latres E, Baumhueter S, Lai VK, Nunez L, Clarke BA, Poueymirou WT, Panaro FJ, Na E, Dharmarajan K (2001). Identification of ubiquitin ligases required for skeletal muscle atrophy. Science.

[47] Huang Z, Zhu J, Ma W, Sun H (2018). Strategies and potential therapeutic agents to counter skeletal muscle atrophy. Biotarget.

[48] Yoshida T, Delafontaine P (1970). Mechanisms of IGF-1-mediated regulation of skeletal muscle hypertrophy and atrophy. Cells.

[49] Fernando R, Drescher C, Nowotny K, Grune T, Castro JP (2019). Impaired proteostasis during skeletal muscle aging. Free Radic Biol Med.

[50] Wang W, Shen D, Zhang L, Ji Y, Xu L, Chen Z, Shen Y, Gong L, Zhang Q, Shen M (2021). SKP-SC-EVs mitigate denervated muscle atrophy by inhibiting oxidative stress and inflammation and improving microcirculation. Antioxidants.

[51] Fernando R, Castro JP, Flore T, Deubel S, Grune T, Ott C (2020). Age-related maintenance of the autophagy-lysosomal system is dependent on skeletal muscle type. Oxid Med Cell Longev.

[52] Sandri M (2010). Autophagy in skeletal muscle. FEBS Lett.

[53] Triolo M, Slavin M, Moradi N, Hood DA (2022). Time-dependent changes in autophagy, mitophagy and lysosomes in skeletal muscle during denervation-induced disuse. J Physiol.

[54] Singh A, Yadav A, Phogat J, Dabur R (2022). Dynamics and interplay between autophagy and ubiquitin-proteasome system coordination in skeletal muscle atrophy. Curr Mol Pharmacol.

[55] Baar K, Nader G, Bodine S (2006). Resistance exercise, muscle loading/unloading and the control of muscle mass. Essays Biochem.

[56] Talbert EE, Smuder AJ, Min K, Kwon OS, Powers SK (1985). Calpain and caspase-3 play required roles in immobilization-induced limb muscle atrophy. J Appl Physiol.

[57] Smuder AJ, Kavazis AN, Hudson MB, Nelson WB, Powers SK (2010). Oxidation enhances myofibrillar protein degradation via calpain and caspase-3. Free Radic Biol Med.

[58] Komatsu R, Okazaki T, Ebihara S, Kobayashi M, Tsukita Y, Nihei M, Sugiura H, Niu K, Ebihara T, Ichinose M (2018). Aspiration pneumonia induces muscle atrophy in the respiratory, skeletal, and swallowing systems. J Cachexia Sarcopenia Muscle.

[59] Jang YC, Rodriguez K, Lustgarten MS, Muller FL, Bhattacharya A, Pierce A, Choi JJ, Lee NH, Chaudhuri A, Richardson AG, Van Remmen H (2020). Superoxide-mediated oxidative stress accelerates skeletal muscle atrophy by synchronous activation of proteolytic systems. Geroscience.

[60] White JP (2021). Amino acid trafficking and skeletal muscle protein synthesis: a case of supply and demand. Front Cell Dev Biol.

[61] Mirzoev TM (2020). Skeletal muscle recovery from disuse atrophy: protein turnover signaling and strategies for accelerating muscle regrowth. Int J Mol Sci.

[62] Gatica D, Klionsky DJ (2017). New insights into MTORC1 amino acid sensing and activation. Biotarget.

[63] Rudrappa SS, Wilkinson DJ, Greenhaff PL, Smith K, Idris I, Atherton PJ (2016). Human skeletal muscle disuse atrophy: effects on muscle protein synthesis, breakdown, and insulin resistance-a qualitative review. Front Physiol.

[64] Baehr LM, West DWD, Marshall AG, Marcotte GR, Baar K, Bodine SC (1985). Muscle-specific and age-related changes in protein synthesis and protein degradation in response to hindlimb unloading in rats. J Appl Physiol.

[65] Tokinoya K, Shirai T, Ota Y, Takemasa T, Takekoshi K (2020). Denervation-induced muscle atrophy suppression in renalase-deficient mice via increased protein synthesis. Physiol Rep.

[66] Feng L, Li B, Xi Y, Cai M, Tian Z (2022). Aerobic exercise and resistance exercise alleviate skeletal muscle atrophy through IGF-1/IGF-1R-PI3K/Akt pathway in mice with myocardial infarction. Am J Physiol Cell Physiol.

[67] Fukada SI, Akimoto T, Sotiropoulos A (2020). Role of damage and management in muscle hypertrophy: different behaviors of muscle stem cells in regeneration and hypertrophy. Biochim Biophys Acta Mol Cell Res.

[68] Joseph J, Doles JD (2021). Disease-associated metabolic alterations that impact satellite cells and muscle regeneration: perspectives and therapeutic outlook. Nutr Metab (Lond).

[69] Romagnoli C, Iantomasi T, Brandi ML (2021). Available In vitro models for human satellite cells from skeletal muscle. Int J Mol Sci.

[70] Wosczyna MN, Konishi CT, Perez Carbajal EE, Wang TT, Walsh RA, Gan Q, Wagner MW, Rando TA (2019). Mesenchymal stromal cells are required for regeneration and homeostatic maintenance of skeletal muscle. Cell Rep.

[71] Wong A, Pomerantz JH (2019). The role of muscle stem cells in regeneration and recovery after denervation: a review. Plast Reconstr Surg.

[72] Tachtsis B, Camera D, Lacham-Kaplan O (2018). Potential roles of n-3 PUFAs during skeletal muscle growth and regeneration. Nutrients.

[73] Ebadi M, Tsien C, Bhanji RA, Dunichand-Hoedl AR, Rider E, Motamedrad M, Mazurak VC, Baracos V, Montano-Loza AJ (2022). Skeletal muscle pathological fat infiltration (myosteatosis) is associated with higher mortality in patients with cirrhosis. Cells.

[74] Gonzalez D, Contreras O, Rebolledo DL, Espinoza JP, van Zundert B, Brandan E (2017). ALS skeletal muscle shows enhanced TGF-beta signaling, fibrosis and induction of fibro/adipogenic progenitor markers. PLoS ONE.

[75] Engelke K, Ghasemikaram M, Chaudry O, Uder M, Nagel AM, Jakob F, Kemmler W (2022). The effect of ageing on fat infiltration of thigh and paraspinal muscles in men. Aging Clin Exp Res.

[76] Wu H, Jang J, Dridi S, Ferrando AA, Wolfe RR, Kim IY, Baum JI (2020). Net protein balance correlates with expression of autophagy, mitochondrial biogenesis, and fat metabolism-related genes in skeletal muscle from older adults. Physiol Rep.

[77] Shang GK, Han L, Wang ZH, Liu YP, Yan SB, Sai WW, Wang D, Li YH, Zhang W, Zhong M (2020). Sarcopenia is attenuated by TRB3 knockout in aging mice via the alleviation of atrophy and fibrosis of skeletal muscles. J Cachexia Sarcopenia Muscle.

[78] Mendias CL, Gumucio JP, Davis ME, Bromley CW, Davis CS, Brooks SV (2012). Transforming growth factor-beta induces skeletal muscle atrophy and fibrosis through the induction of atrogin-1 and scleraxis. Muscle Nerve.

[79] Li CW, Yu K, Shyh-Chang N, Jiang Z, Liu T, Ma S, Luo L, Guang L, Liang K, Ma W (2022). Pathogenesis of sarcopenia and the relationship with fat mass: descriptive review. J Cachexia Sarcopenia Muscle.

[80] Lyu AK, Zhu SY, Chen JL, Zhao YX, Pu D, Luo C, Lyu Q, Fan Z, Sun Y, Wu J (2019). Inhibition of TLR9 attenuates skeletal muscle fibrosis in aged sarcopenic mice via the p53/SIRT1 pathway. Exp Gerontol.

[81] Horii N, Uchida M, Hasegawa N, Fujie S, Oyanagi E, Yano H, Hashimoto T, Iemitsu M (2018). Resistance training prevents muscle fibrosis and atrophy via down-regulation of C1q-induced Wnt signaling in senescent mice. FASEB J.

[82] Wang H, Wang B, Wei J, Zheng Z, Su J, Bian C, Xin Y, Jiang X (2022). Sulforaphane regulates Nrf2-mediated antioxidant activity and downregulates TGF-beta1/Smad pathways to prevent radiation-induced muscle fibrosis. Life Sci.

[83] Qu Z, Zhou S, Li P, Liu C, Yuan B, Zhang S, Liu A (2021). Natural products and skeletal muscle health. J Nutr Biochem.

[84] Tomimatsu T, Miyazaki J, Kano Y, Kobayashi T (2017). Photothermal imaging of skeletal muscle mitochondria. Biomed Opt Express.

[85] Romanello V, Sandri M (2010). Mitochondrial biogenesis and fragmentation as regulators of muscle protein degradation. Curr Hypertens Rep.

[86] Tamura Y, Kawano S, Endo T (2020). Lipid homeostasis in mitochondria. Biol Chem.

[87] Alfarouk KO, Alhoufie STS, Hifny A, Schwartz L, Alqahtani AS, Ahmed SBM, Alqahtani AM, Alqahtani SS, Muddathir AK, Ali H (2021). Of mitochondrion and COVID-19. J Enzyme Inhib Med Chem.

[88] Maglioni S, Ventura N (2016). C elegans as a model organism for human mitochondrial associated disorders. Mitochondrion.

[89] Whitley BN, Engelhart EA, Hoppins S (2019). Mitochondrial dynamics and their potential as a therapeutic target. Mitochondrion.

[90] Rodrigues T, Ferraz LS (2020). Therapeutic potential of targeting mitochondrial dynamics in cancer. Biochem Pharmacol.

[91] Xie LL, Shi F, Tan Z, Li Y, Bode AM, Cao Y (2018). Mitochondrial network structure homeostasis and cell death. Cancer Sci.

[92] Kleele T, Rey T, Winter J, Zaganelli S, Mahecic D, Perreten Lambert H, Ruberto FP, Nemir M, Wai T, Pedrazzini T, Manley S (2021). Distinct fission signatures predict mitochondrial degradation or biogenesis. Nature.

[93] Li PA, Hou X, Hao S (2017). Mitochondrial biogenesis in neurodegeneration. J Neurosci Res.

[94] Popov LD (2020). Mitochondrial biogenesis: an update. J Cell Mol Med.

[95] Liu D, Fan YB, Tao XH, Pan WL, Wu YX, Wang XH, He YQ, Xiao WF, Li YS (2021). Mitochondrial quality control in sarcopenia: updated overview of mechanisms and interventions. Aging Dis.

[96] Yang X, Ji Y, Wang W, Zhang L, Chen Z, Yu M, Shen Y, Ding F, Gu X, Sun H (2021). Amyotrophic lateral sclerosis: molecular mechanisms, biomarkers, and therapeutic strategies. Antioxidants.

[97] Bouchez C, Devin A (2019). Mitochondrial biogenesis and mitochondrial reactive oxygen species (ROS): a complex relationship regulated by the camp/pka signaling pathway. Cells.

[98] Xia Q, Huang X, Huang J, Zheng Y, March ME, Li J, Wei Y (2021). The role of autophagy in skeletal muscle diseases. Front Physiol.

[99] Zhao Y, Feng X, Li B, Sha J, Wang C, Yang T, Cui H, Fan H (2020). Dexmedetomidine protects against lipopolysaccharide-induced acute kidney injury by enhancing autophagy through inhibition of the PI3K/AKT/mTOR pathway. Front Pharmacol.

[100] Kim Y, Triolo M, Hood DA (2017). Impact of aging and exercise on mitochondrial quality control in skeletal muscle. Oxid Med Cell Longev.

[101] Galluzzi L, Baehrecke EH, Ballabio A, Boya P, Bravo-San Pedro JM, Cecconi F, Choi AM, Chu CT, Codogno P, Colombo MI (2017). Molecular definitions of autophagy and related processes. EMBO J.

[102] Murata D, Arai K, Iijima M, Sesaki H (2020). Mitochondrial division, fusion and degradation. J Biochem.

[103] He C, Bassik MC, Moresi V, Sun K, Wei Y, Zou Z, An Z, Loh J, Fisher J, Sun Q (2012). Exercise-induced BCL2-regulated autophagy is required for muscle glucose homeostasis. Nature.

[104] Tian L, Cao W, Yue R, Yuan Y, Guo X, Qin D, Xing J, Wang X (2019). Pretreatment with Tilianin improves mitochondrial energy metabolism and oxidative stress in rats with myocardial ischemia/reperfusion injury via AMPK/SIRT1/PGC-1 alpha signaling pathway. J Pharmacol Sci.

[105] Salt IP, Hardie DG (2017). AMP-activated protein kinase: an ubiquitous signaling pathway with key roles in the cardiovascular system. Circ Res.

[106] Canto C, Gerhart-Hines Z, Feige JN, Lagouge M, Noriega L, Milne JC, Elliott PJ, Puigserver P, Auwerx J (2009). AMPK regulates energy expenditure by modulating NAD+ metabolism and SIRT1 activity. Nature.

[107] Guo A, Li K, Xiao Q (2020). Fibroblast growth factor 19 alleviates palmitic acid-induced mitochondrial dysfunction and oxidative stress via the AMPK/PGC-1alpha pathway in skeletal muscle. Biochem Biophys Res Commun.

[108] Kou X, Li J, Liu X, Yang X, Fan J, Chen N (2017). Ampelopsin attenuates the atrophy of skeletal muscle from d-gal-induced aging rats through activating AMPK/SIRT1/PGC-1alpha signaling cascade. Biomed Pharmacother.

[109] Aversa Z, Zhang X, Fielding RA, Lanza I, LeBrasseur NK (2019). The clinical impact and biological mechanisms of skeletal muscle aging. Bone.

[110] Shin JE, Park SJ, Ahn SI, Choung SY (2020). Soluble whey protein hydrolysate ameliorates muscle atrophy induced by immobilization via regulating the PI3K/Akt pathway in C57BL/6 mice. Nutrients.

[111] Munson MJ, Ganley IG (2015). MTOR, PIK3C3, and autophagy: signaling the beginning from the end. Autophagy.

[112] Bhardwaj G, Penniman CM, Jena J, Suarez Beltran PA, Foster C, Poro K, Junck TL, Hinton AO, Souvenir R, Fuqua JD (2021). Insulin and IGF-1 receptors regulate complex I-dependent mitochondrial bioenergetics and supercomplexes via FoxOs in muscle. J Clin Invest.

[113] Ding Y, Li J, Liu Z, Liu H, Li H, Li Z (2017). IGF-1 potentiates sensory innervation signalling by modulating the mitochondrial fission/fusion balance. Sci Rep.

[114] Uemichi K, Shirai T, Hanakita H, Takemasa T (2021). Effect of mechanistic/mammalian target of rapamycin complex 1 on mitochondrial dynamics during skeletal muscle hypertrophy. Physiol Rep.

[115] Tang G, Du Y, Guan H, Jia J, Zhu N, Shi Y, Rong S, Yuan W (2022). Butyrate ameliorates skeletal muscle atrophy in diabetic nephropathy by enhancing gut barrier function and FFA2-mediated PI3K/Akt/mTOR signals. Br J Pharmacol.

[116] Yin D, Lin D, Xie Y, Gong A, Jiang P, Wu J (2022). Neuregulin-1beta alleviates sepsis-induced skeletal muscle atrophy by inhibiting autophagy via AKT/mTOR signaling pathway in rats. Shock.

[117] Mammucari C, Milan G, Romanello V, Masiero E, Rudolf R, Del Piccolo P, Burden SJ, Di Lisi R, Sandri C, Zhao J (2007). FoxO3 controls autophagy in skeletal muscle in vivo. Cell Metab.

[118] Zhao J, Brault JJ, Schild A, Cao P, Sandri M, Schiaffino S, Lecker SH, Goldberg AL (2007). FoxO3 coordinately activates protein degradation by the autophagic/lysosomal and proteasomal pathways in atrophying muscle cells. Cell Metab.

[119] Sandri M (2013). Protein breakdown in muscle wasting: role of autophagy-lysosome and ubiquitin-proteasome. Int J Biochem Cell Biol.

[120] Munoz-Canoves P, Scheele C, Pedersen BK, Serrano AL (2013). Interleukin-6 myokine signaling in skeletal muscle: a double-edged sword?. FEBS J.

[121] Sala D, Cunningham TJ, Stec MJ, Etxaniz U, Nicoletti C, Dall'Agnese A, Puri PL, Duester G, Latella L, Sacco A (2019). The Stat3-Fam3a axis promotes muscle stem cell myogenic lineage progression by inducing mitochondrial respiration. Nat Commun.

[122] Min K, Lawan A, Bennett AM (2017). Loss of MKP-5 promotes myofiber survival by activating STAT3/Bcl-2 signaling during regenerative myogenesis. Skelet Muscle.

[123] Huot JR, Novinger LJ, Pin F, Bonetto A (2020). HCT116 colorectal liver metastases exacerbate muscle wasting in a mouse model for the study of colorectal cancer cachexia. Dis Model Mech.

[124] Abrigo J, Campos F, Simon F, Riedel C, Cabrera D, Vilos C, Cabello-Verrugio C (2018). TGF-beta requires the activation of canonical and non-canonical signalling pathways to induce skeletal muscle atrophy. Biol Chem.

[125] Roy A, Sharma AK, Nellore K, Narkar VA, Kumar A (2020). TAK1 preserves skeletal muscle mass and mitochondrial function through redox homeostasis. FASEB Bioadv.

[126] Roy A, Kumar A (2022). Supraphysiological activation of TAK1 promotes skeletal muscle growth and mitigates neurogenic atrophy. Nat Commun.

[127] Lin L, Hou G, Han D, Yin Y, Kang J, Wang Q (2019). Ursolic acid alleviates airway-vessel remodeling and muscle consumption in cigarette smoke-induced emphysema rats. BMC Pulm Med.

[128] Yadav H, Quijano C, Kamaraju AK, Gavrilova O, Malek R, Chen W, Zerfas P, Zhigang D, Wright EC, Stuelten C (2011). Protection from obesity and diabetes by blockade of TGF-beta/Smad3 signaling. Cell Metab.

[129] Bohm A, Hoffmann C, Irmler M, Schneeweiss P, Schnauder G, Sailer C, Schmid V, Hudemann J, Machann J, Schick F (2016). TGF-beta contributes to impaired exercise response by suppression of mitochondrial key regulators in skeletal muscle. Diabetes.

[130] Bhatnagar S, Mittal A, Gupta SK, Kumar A (2012). TWEAK causes myotube atrophy through coordinated activation of ubiquitin-proteasome system, autophagy, and caspases. J Cell Physiol.

[131] Ge X, Tang P, Rong Y, Jiang D, Lu X, Ji C, Wang J, Huang C, Duan A, Liu Y (2021). Exosomal miR-155 from M1-polarized macrophages promotes EndoMT and impairs mitochondrial function via activating NF-kappaB signaling pathway in vascular endothelial cells after traumatic spinal cord injury. Redox Biol.

[132] Nisr RB, Shah DS, Ganley IG, Hundal HS (2019). Proinflammatory NFkB signalling promotes mitochondrial dysfunction in skeletal muscle in response to cellular fuel overloading. Cell Mol Life Sci.

[133] Valentine JM, Li ME, Shoelson SE, Zhang N, Reddick RL, Musi N (2020). NFkappaB regulates muscle development and mitochondrial function. J Gerontol A Biol Sci Med Sci.

[134] Seo DY, Lee SR, Kim N, Ko KS, Rhee BD, Han J (2016). Age-related changes in skeletal muscle mitochondria: the role of exercise. Integr Med Res.

[135] Favaro G, Romanello V, Varanita T, Andrea Desbats M, Morbidoni V, Tezze C, Albiero M, Canato M, Gherardi G, De Stefani D (2019). DRP1-mediated mitochondrial shape controls calcium homeostasis and muscle mass. Nat Commun.

[136] Tezze C, Romanello V, Desbats MA, Fadini GP, Albiero M, Favaro G, Ciciliot S, Soriano ME, Morbidoni V, Cerqua C (2017). Age-associated loss of OPA1 in muscle impacts muscle mass, metabolic homeostasis, systemic inflammation, and epithelial senescence. Cell Metab.

[137] Bulthuis EP, Adjobo-Hermans MJW, Willems P, Koopman WJH (2019). Mitochondrial morphofunction in mammalian cells. Antioxid Redox Signal.

[138] Jornayvaz FR, Shulman GI (2010). Regulation of mitochondrial biogenesis. Essays Biochem.

[139] Herbst A, Wanagat J, Cheema N, Widjaja K, McKenzie D, Aiken JM (2016). Latent mitochondrial DNA deletion mutations drive muscle fiber loss at old age. Aging Cell.

[140] McKenzie D, Bua E, McKiernan S, Cao Z, Aiken JM, Jonathan W (2002). Mitochondrial DNA deletion mutations: a causal role in sarcopenia. Eur J Biochem.

[141] Lagouge M, Larsson NG (2013). The role of mitochondrial DNA mutations and free radicals in disease and ageing. J Intern Med.

[142] Bilodeau PA, Coyne ES, Wing SS (2016). The ubiquitin proteasome system in atrophying skeletal muscle: roles and regulation. Am J Physiol Cell Physiol.

[143] Carmignac V, Svensson M, Korner Z, Elowsson L, Matsumura C, Gawlik KI, Allamand V, Durbeej M (2011). Autophagy is increased in laminin alpha2 chain-deficient muscle and its inhibition improves muscle morphology in a mouse model of MDC1A. Hum Mol Genet.

[144] Masiero E, Agatea L, Mammucari C, Blaauw B, Loro E, Komatsu M, Metzger D, Reggiani C, Schiaffino S, Sandri M (2009). Autophagy is required to maintain muscle mass. Cell Metab.

[145] Seabright AP, Fine NHF, Barlow JP, Lord SO, Musa I, Gray A, Bryant JA, Banzhaf M, Lavery GG, Hardie DG (2020). AMPK activation induces mitophagy and promotes mitochondrial fission while activating TBK1 in a PINK1-Parkin independent manner. FASEB J.

[146] Munteanu I, Kalimo H, Saraste A, Nishino I, Minassian BA (2017). Cardiac autophagic vacuolation in severe X-linked myopathy with excessive autophagy. Neuromuscul Disord.

[147] Fernandes SA, Almeida CF, Souza LS, Lazar M, Onofre-Oliveira P, Yamamoto GL, Nogueira L, Tasaki LY, Cardoso RR, Pavanello RCM (2020). Altered in vitro muscle differentiation in X-linked myopathy with excessive autophagy. Dis Model Mech.

[148] O'Neill BT, Bhardwaj G, Penniman CM, Krumpoch MT, Suarez Beltran PA, Klaus K, Poro K, Li M, Pan H, Dreyfuss JM (2019). FoxO transcription factors are critical regulators of diabetes-related muscle atrophy. Diabetes.

[149] Huang DD, Yan XL, Fan SD, Chen XY, Yan JY, Dong QT, Chen WZ, Liu NX, Chen XL, Yu Z (2020). Nrf2 deficiency promotes the increasing trend of autophagy during aging in skeletal muscle: a potential mechanism for the development of sarcopenia. Aging.

[150] Rodney GG, Pal R, Abo-Zahrah R (2016). Redox regulation of autophagy in skeletal muscle. Free Radic Biol Med.

[151] Muller FL, Song W, Jang YC, Liu Y, Sabia M, Richardson A, Van Remmen H (2007). Denervation-induced skeletal muscle atrophy is associated with increased mitochondrial ROS production. Am J Physiol Regul Integr Comp Physiol.

[152] Yang B, Yang X, Sun X, Shi J, Shen Y, Chen R (2022). IL-6 Deficiency attenuates skeletal muscle atrophy by inhibiting mitochondrial ROS production through the upregulation of PGC-1alpha in septic mice. Oxid Med Cell Longev.

[153] Shally A, McDonagh B (2020). The redox environment and mitochondrial dysfunction in age-related skeletal muscle atrophy. Biogerontology.

[154] Groening P, Huang Z, La Gamma EF, Levy RJ (2011). Glutamine restores myocardial cytochrome C oxidase activity and improves cardiac function during experimental sepsis. JPEN J Parenter Enteral Nutr.

[155] Larsen S, Nielsen J, Hansen CN, Nielsen LB, Wibrand F, Stride N, Schroder HD, Boushel R, Helge JW, Dela F, Hey-Mogensen M (2012). Biomarkers of mitochondrial content in skeletal muscle of healthy young human subjects. J Physiol.

[156] Yeo D, Kang C, Gomez-Cabrera MC, Vina J, Ji LL (2019). Intensified mitophagy in skeletal muscle with aging is downregulated by PGC-1alpha overexpression in vivo. Free Radic Biol Med.

[157] Kitaoka Y, Miyazaki M, Kikuchi S (2021). Voluntary exercise prevents abnormal muscle mitochondrial morphology in cancer cachexia mice. Physiol Rep.

[158] Bravo-Sagua R, Parra V, Lopez-Crisosto C, Diaz P, Quest AF, Lavandero S (2017). Calcium transport and signaling in mitochondria. Compr Physiol.

[159] Gherardi G, Di Marco G, Rizzuto R, Mammucari C (2019). Crosstalk between mitochondrial Ca(2+) uptake and autophagy in skeletal muscle. Oxid Med Cell Longev.

[160] Fan M, Zhang J, Tsai CW, Orlando BJ, Rodriguez M, Xu Y, Liao M, Tsai MF, Feng L (2020). Structure and mechanism of the mitochondrial Ca(2+) uniporter holocomplex. Nature.

[161] Sancak Y, Markhard AL, Kitami T, Kovacs-Bogdan E, Kamer KJ, Udeshi ND, Carr SA, Chaudhuri D, Clapham DE, Li AA (2013). EMRE is an essential component of the mitochondrial calcium uniporter complex. Science.

[162] Peng TI, Jou MJ (2010). Oxidative stress caused by mitochondrial calcium overload. Ann N Y Acad Sci.

[163] Mammucari C, Gherardi G, Zamparo I, Raffaello A, Boncompagni S, Chemello F, Cagnin S, Braga A, Zanin S, Pallafacchina G (2015). The mitochondrial calcium uniporter controls skeletal muscle trophism in vivo. Cell Rep.

[164] Logan CV, Szabadkai G, Sharpe JA, Parry DA, Torelli S, Childs AM, Kriek M, Phadke R, Johnson CA, Roberts NY (2014). Loss-of-function mutations in MICU1 cause a brain and muscle disorder linked to primary alterations in mitochondrial calcium signaling. Nat Genet.

[165] Tsai CW, Rodriguez MX, Van Keuren AM, Phillips CB, Shushunov HM, Lee JE, Garcia AM, Ambardekar AV, Cleveland JC, Reisz JA (2022). Mechanisms and significance of tissue-specific MICU regulation of the mitochondrial calcium uniporter complex. Mol Cell.

[166] Liu JC, Syder NC, Ghorashi NS, Willingham TB, Parks RJ, Sun J, Fergusson MM, Liu J, Holmstrom KM, Menazza S (2020). EMRE is essential for mitochondrial calcium uniporter activity in a mouse model. JCI Insight.

[167] Kim SH, Kim H (2018). Inhibitory effect of astaxanthin on oxidative stress-induced mitochondrial dysfunction-a mini-review. Nutrients.

[168] Robichaux DJ, Harata M, Murphy E, Karch J (2023). Mitochondrial permeability transition pore-dependent necrosis. J Mol Cell Cardiol.

[169] Alway SE, Mohamed JS, Myers MJ (2017). Mitochondria initiate and regulate sarcopenia. Exerc Sport Sci Rev.

[170] Basse AL, Agerholm M, Farup J, Dalbram E, Nielsen J, Ortenblad N, Altintas A, Ehrlich AM, Krag T, Bruzzone S (2021). Nampt controls skeletal muscle development by maintaining Ca(2+) homeostasis and mitochondrial integrity. Mol Metab.

[171] Patel P, Mendoza A, Robichaux DJ, Wang MC, Wehrens XHT, Karch J (2021). Inhibition of the anti-apoptotic Bcl-2 family by BH3 mimetics sensitize the mitochondrial permeability transition pore through bax and bak. Front Cell Dev Biol.

[172] Spendiff S, Vuda M, Gouspillou G, Aare S, Perez A, Morais JA, Jagoe RT, Filion ME, Glicksman R, Kapchinsky S (2016). Denervation drives mitochondrial dysfunction in skeletal muscle of octogenarians. J Physiol.

[173] Skinner SK, Solania A, Wolan DW, Cohen MS, Ryan TE, Hepple RT (2021). Mitochondrial permeability transition causes mitochondrial reactive oxygen species- and caspase 3-dependent atrophy of single adult mouse skeletal muscle fibers. Cells.

[174] Gebert N, Joshi AS, Kutik S, Becker T, McKenzie M, Guan XL, Mooga VP, Stroud DA, Kulkarni G, Wenk MR (2009). Mitochondrial cardiolipin involved in outer-membrane protein biogenesis: implications for barth syndrome. Curr Biol.

[175] Chu CT, Ji J, Dagda RK, Jiang JF, Tyurina YY, Kapralov AA, Tyurin VA, Yanamala N, Shrivastava IH, Mohammadyani D (2013). Cardiolipin externalization to the outer mitochondrial membrane acts as an elimination signal for mitophagy in neuronal cells. Nat Cell Biol.

[176] Pan L, Xie W, Fu X, Lu W, Jin H, Lai J, Zhang A, Yu Y, Li Y, Xiao W (2021). Inflammation and sarcopenia: a focus on circulating inflammatory cytokines. Exp Gerontol.

[177] Hughes DC, Baehr LM, Waddell DS, Sharples AP, Bodine SC (2022). Ubiquitin ligases in longevity and aging skeletal muscle. Int J Mol Sci.

[178] Migliavacca E, Tay SKH, Patel HP, Sonntag T, Civiletto G, McFarlane C, Forrester T, Barton SJ, Leow MK, Antoun E (2019). Mitochondrial oxidative capacity and NAD(+) biosynthesis are reduced in human sarcopenia across ethnicities. Nat Commun.

[179] Bellanti F, Lo Buglio A, Vendemiale G (2021). Mitochondrial Impairment in Sarcopenia. Biology.

[180] Memme JM, Slavin M, Moradi N, Hood DA (2021). Mitochondrial bioenergetics and turnover during chronic muscle disuse. Int J Mol Sci.

[181] Rosa-Caldwell ME, Lim S, Haynie WS, Brown JL, Lee DE, Dunlap KR, Jansen LT, Washington TA, Wiggs MP, Greene NP (2021). Mitochondrial aberrations during the progression of disuse atrophy differentially affect male and female mice. J Cachexia Sarcopenia Muscle.

[182] Calvani R, Joseph AM, Adhihetty PJ, Miccheli A, Bossola M, Leeuwenburgh C, Bernabei R, Marzetti E (2013). Mitochondrial pathways in sarcopenia of aging and disuse muscle atrophy. Biol Chem.

[183] Yokokawa T, Mori R, Suga T, Isaka T, Hayashi T, Fujita S (2020). Muscle denervation reduces mitochondrial biogenesis and mitochondrial translation factor expression in mice. Biochem Biophys Res Commun.

[184] Yang X, Xue P, Chen H, Yuan M, Kang Y, Duscher D, Machens HG, Chen Z (2020). Denervation drives skeletal muscle atrophy and induces mitochondrial dysfunction, mitophagy and apoptosis via miR-142a-5p/MFN1 axis. Theranostics.

[185] Gorgey AS, Witt O, O'Brien L, Cardozo C, Chen Q, Lesnefsky EJ, Graham ZA (2019). Mitochondrial health and muscle plasticity after spinal cord injury. Eur J Appl Physiol.

[186] Li Y, Jin H, Chen Y, Huang T, Mi Y, Zou Z (2021). Cancer cachexia: molecular mechanism and pharmacological management. Biochem J.

[187] Dolly A, Dumas JF, Servais S (2020). Cancer cachexia and skeletal muscle atrophy in clinical studies: what do we really know?. J Cachexia Sarcopenia Muscle.

[188] Zhang Y, Wang J, Wang X, Gao T, Tian H, Zhou D, Zhang L, Li G, Wang X (2020). The autophagic-lysosomal and ubiquitin proteasome systems are simultaneously activated in the skeletal muscle of gastric cancer patients with cachexia. Am J Clin Nutr.

[189] van der Ende M, Grefte S, Plas R, Meijerink J, Witkamp RF, Keijer J, van Norren K (2018). Mitochondrial dynamics in cancer-induced cachexia. Biochim Biophys Acta Rev Cancer.

[190] Penna F, Ballaro R, Martinez-Cristobal P, Sala D, Sebastian D, Busquets S, Muscaritoli M, Argiles JM, Costelli P, Zorzano A (2019). Autophagy exacerbates muscle wasting in cancer cachexia and impairs mitochondrial function. J Mol Biol.

[191] Irazabal MV, Torres VE (2020). Reactive oxygen species and redox signaling in chronic kidney disease. Cells.

[192] Fontecha-Barriuso M, Martin-Sanchez D, Martinez-Moreno JM, Monsalve M, Ramos AM, Sanchez-Nino MD, Ruiz-Ortega M, Ortiz A, Sanz AB (2020). The role of PGC-1alpha and mitochondrial biogenesis in kidney diseases. Biomolecules.

[193] Jiang M, Bai M, Lei J, Xie Y, Xu S, Jia Z, Zhang A (2020). Mitochondrial dysfunction and the AKI-to-CKD transition. Am J Physiol Renal Physiol.

[194] Su Z, Klein JD, Du J, Franch HA, Zhang L, Hassounah F, Hudson MB, Wang XH (2017). Chronic kidney disease induces autophagy leading to dysfunction of mitochondria in skeletal muscle. Am J Physiol Renal Physiol.

[195] Wang M, Hu R, Wang Y, Liu L, You H, Zhang J, Wu X, Pei T, Wang F, Lu L (2019). Atractylenolide III attenuates muscle wasting in chronic kidney disease via the oxidative stress-mediated PI3K/AKT/mTOR pathway. Oxid Med Cell Longev.

[196] Zhang YY, Gu LJ, Huang J, Cai MC, Yu HL, Zhang W, Bao JF, Yuan WJ (2019). CKD autophagy activation and skeletal muscle atrophy-a preliminary study of mitophagy and inflammation. Eur J Clin Nutr.

[197] Wang D, Sun H, Song G, Yang Y, Zou X, Han P, Li S (2018). Resveratrol improves muscle atrophy by modulating mitochondrial quality control in STZ-induced diabetic mice. Mol Nutr Food Res.

[198] Pileggi CA, Parmar G, Harper ME (2021). The lifecycle of skeletal muscle mitochondria in obesity. Obes Rev.

[199] Andres-Hernando A, Lanaspa MA, Kuwabara M, Orlicky DJ, Cicerchi C, Bales E, Garcia GE, Roncal-Jimenez CA, Sato Y, Johnson RJ (2019). Obesity causes renal mitochondrial dysfunction and energy imbalance and accelerates chronic kidney disease in mice. Am J Physiol Renal Physiol.

[200] Kim KW, Baek MO, Yoon MS, Son KH (2021). Deterioration of mitochondrial function in the human intercostal muscles differs among individuals with sarcopenia, obesity, and sarcopenic obesity. Clin Nutr.

[201] Dantas WS, Zunica ERM, Heintz EC, Vandanmagsar B, Floyd ZE, Yu Y, Fujioka H, Hoppel CL, Belmont KP, Axelrod CL, Kirwan JP (2022). Mitochondrial uncoupling attenuates sarcopenic obesity by enhancing skeletal muscle mitophagy and quality control. J Cachexia Sarcopenia Muscle.

[202] Yang X, Ji Y, Wang W, Zhang L, Chen Z, Yu M, Shen Y, Ding F, Gu X, Sun H (2021). Amyotrophic lateral sclerosis: molecular mechanisms, biomarkers, and therapeutic strategies. Antioxidants.

[203] Sun H, Li M, Ji Y, Zhu J, Chen Z, Zhang L, Deng C, Cheng Q, Wang W, Shen Y, Shen D (2022). Identification of regulatory factors and prognostic markers in amyotrophic lateral sclerosis. Antioxidants.

[204] Zuo X, Zhou J, Li Y, Wu K, Chen Z, Luo Z, Zhang X, Liang Y, Esteban MA, Zhou Y, Fu XD (2021). TDP-43 aggregation induced by oxidative stress causes global mitochondrial imbalance in ALS. Nat Struct Mol Biol.

[205] Smith EF, Shaw PJ, De Vos KJ (2019). The role of mitochondria in amyotrophic lateral sclerosis. Neurosci Lett.

[206] Singh A, Kukreti R, Saso L, Kukreti S (2019). Oxidative stress: a key modulator in neurodegenerative diseases. Molecules.

[207] Falabella M, Vernon HJ, Hanna MG, Claypool SM, Pitceathly RDS (2021). Cardiolipin, mitochondria, and neurological disease. Trends Endocrinol Metab.

[208] Bora G, Hensel N, Rademacher S, Koyunoglu D, Sunguroglu M, Aksu-Menges E, Balci-Hayta B, Claus P, Erdem-Yurter H (2021). Microtubule-associated protein 1B dysregulates microtubule dynamics and neuronal mitochondrial transport in spinal muscular atrophy. Hum Mol Genet.

[209] Chaytow H, Huang YT, Gillingwater TH, Faller KME (2018). The role of survival motor neuron protein (SMN) in protein homeostasis. Cell Mol Life Sci.

[210] Singh NN, Hoffman S, Reddi PP, Singh RN (2021). Spinal muscular atrophy: Broad disease spectrum and sex-specific phenotypes. Biochim Biophys Acta Mol Basis Dis.

[211] Miller N, Shi H, Zelikovich AS, Ma YC (2016). Motor neuron mitochondrial dysfunction in spinal muscular atrophy. Hum Mol Genet.

[212] Hashizume A, Fischbeck KH, Pennuto M, Fratta P, Katsuno M (2020). Disease mechanism, biomarker and therapeutics for spinal and bulbar muscular atrophy (SBMA). J Neurol Neurosurg Psychiatry.

[213] Chivet M, Marchioretti C, Pirazzini M, Piol D, Scaramuzzino C, Polanco MJ, Romanello V, Zuccaro E, Parodi S, D'Antonio M (2020). Polyglutamine-expanded androgen receptor alteration of skeletal muscle homeostasis and myonuclear aggregation are affected by sex. Age Muscle Metabo Cells.

[214] Pourshafie N, Masati E, Lopez A, Bunker E, Snyder A, Edwards NA, Winkelsas AM, Fischbeck KH, Grunseich C (2022). Altered SYNJ2BP-mediated mitochondrial-ER contacts in motor neuron disease. Neurobiol Dis.

[215] Pourshafie N, Masati E, Bunker E, Nickolls AR, Thepmankorn P, Johnson K, Feng X, Ekins T, Grunseich C, Fischbeck KH (2020). Linking epigenetic dysregulation, mitochondrial impairment, and metabolic dysfunction in SBMA motor neurons. JCI Insight.

[216] Zablocka B, Gorecki DC, Zablocki K (2021). Disrupted calcium homeostasis in duchenne muscular dystrophy: a common mechanism behind diverse consequences. Int J Mol Sci.

[217] Chang M, Cai Y, Gao Z, Chen X, Liu B, Zhang C, Yu W, Cao Q, Shen Y, Yao X (2023). Duchenne muscular dystrophy: pathogenesis and promising therapies. J Neurol.

[218] Budzinska M, Zimna A, Kurpisz M (2021). The role of mitochondria in Duchenne muscular dystrophy. J Physiol Pharmacol.

[219] Reid AL, Alexander MS (2021). The interplay of mitophagy and inflammation in duchenne muscular dystrophy. Life (Basel).

[220] van Westering TL, Betts CA, Wood MJ (2015). Current understanding of molecular pathology and treatment of cardiomyopathy in duchenne muscular dystrophy. Molecules.

[221] Zielonka J, Joseph J, Sikora A, Hardy M, Ouari O, Vasquez-Vivar J, Cheng G, Lopez M, Kalyanaraman B (2017). Mitochondria-targeted triphenylphosphonium-based compounds: syntheses, mechanisms of action, and therapeutic and diagnostic applications. Chem Rev.

[222] Lopez-Cervantes SP, Sanchez NS, Calahorra M, Mena-Montes B, Pedraza-Vazquez G, Hernandez-Alvarez D, Esparza-Perusquia M, Pena A, Lopez-Diazguerrero NE, Alarcon-Aguilar A (2022). Moderate exercise combined with metformin-treatment improves mitochondrial bioenergetics of the quadriceps muscle of old female Wistar rats. Arch Gerontol Geriatr.

[223] Huang Y, Zhu X, Chen K, Lang H, Zhang Y, Hou P, Ran L, Zhou M, Zheng J, Yi L (2019). Resveratrol prevents sarcopenic obesity by reversing mitochondrial dysfunction and oxidative stress via the PKA/LKB1/AMPK pathway. Aging.

[224] Javadov S, Jang S, Rodriguez-Reyes N, Rodriguez-Zayas AE, Soto Hernandez J, Krainz T, Wipf P, Frontera W (2015). Mitochondria-targeted antioxidant preserves contractile properties and mitochondrial function of skeletal muscle in aged rats. Oncotarget.

[225] Pin F, Huot JR, Bonetto A (2022). The mitochondria-targeting agent mitoq improves muscle atrophy, weakness and oxidative metabolism in C26 tumor-bearing mice. Front Cell Dev Biol.

[226] Szeto HH, Liu S (2018). Cardiolipin-targeted peptides rejuvenate mitochondrial function, remodel mitochondria, and promote tissue regeneration during aging. Arch Biochem Biophys.

[227] Semba RD, Moaddel R, Zhang P, Ramsden CE, Ferrucci L (2019). Tetra-linoleoyl cardiolipin depletion plays a major role in the pathogenesis of sarcopenia. Med Hypotheses.

[228] Wong SK, Ima-Nirwana S, Chin KY (2020). Effects of astaxanthin on the protection of muscle health (Review). Exp Ther Med.

[229] Zhang W, You B, Qi D, Qiu L, Ripley-Gonzalez JW, Zheng F, Fu S, Li C, Dun Y, Liu S (2021). Trimetazidine and exercise provide comparable improvements to high fat diet-induced muscle dysfunction through enhancement of mitochondrial quality control. Sci Rep.

[230] Menduti G, Rasa DM, Stanga S, Boido M (2020). Drug screening and drug repositioning as promising therapeutic approaches for spinal muscular atrophy treatment. Front Pharmacol.

[231] Bose C, Alves I, Singh P, Palade PT, Carvalho E, Borsheim E, Jun SR, Cheema A, Boerma M, Awasthi S, Singh SP (2020). Sulforaphane prevents age-associated cardiac and muscular dysfunction through Nrf2 signaling. Aging Cell.

[232] Sun C, Yang C, Xue R, Li S, Zhang T, Pan L, Ma X, Wang L, Li D (1985). Sulforaphane alleviates muscular dystrophy in mdx mice by activation of Nrf2. J Appl Physiol.

[233] Mercken EM, Mitchell SJ, Martin-Montalvo A, Minor RK, Almeida M, Gomes AP, Scheibye-Knudsen M, Palacios HH, Licata JJ, Zhang Y (2014). SRT2104 extends survival of male mice on a standard diet and preserves bone and muscle mass. Aging Cell.

[234] Qaisar R, Bhaskaran S, Ranjit R, Sataranatarajan K, Premkumar P, Huseman K, Van Remmen H (2019). Restoration of SERCA ATPase prevents oxidative stress-related muscle atrophy and weakness. Redox Biol.

[235] Chen LH, Huang SY, Huang KC, Hsu CC, Yang KC, Li LA, Chan CH, Huang HY (2019). Lactobacillus paracasei PS23 decelerated age-related muscle loss by ensuring mitochondrial function in SAMP8 mice. Aging.

[236] Yeon M, Choi H, Chun KH, Lee JH, Jun HS (2022). Gomisin G improves muscle strength by enhancing mitochondrial biogenesis and function in disuse muscle atrophic mice. Biomed Pharmacother.

[237] Kim R, Kim JW, Lee SJ, Bae GU (2022). Ginsenoside Rg3 protects glucocorticoid-induced muscle atrophy in vitro through improving mitochondrial biogenesis and myotube growth. Mol Med Rep.

[238] Jiwan NC, Appell CR, Wang R, Shen CL, Luk HY (2022). Geranylgeraniol supplementation mitigates soleus muscle atrophy via changes in mitochondrial quality in diabetic rats. In Vivo.

[239] Shen S, Liao Q, Liu J, Pan R, Lee SM, Lin L (2019). Myricanol rescues dexamethasone-induced muscle dysfunction via a sirtuin 1-dependent mechanism. J Cachexia Sarcopenia Muscle.

[240] Huang Y, Chen K, Ren Q, Yi L, Zhu J, Zhang Q, Mi M (2018). Dihydromyricetin attenuates dexamethasone-induced muscle atrophy by improving mitochondrial function via the PGC-1alpha pathway. Cell Physiol Biochem.

[241] Yoo A, Jang YJ, Ahn J, Jung CH, Seo HD, Ha TY (2020). Chrysanthemi Zawadskii var latilobum attenuates obesity-induced skeletal muscle atrophy via regulation of PRMTs in skeletal muscle of mice. Int J Mol Sci.

[242] Li Q, Wu J, Huang J, Hu R, You H, Liu L, Wang D, Wei L (2022). Paeoniflorin ameliorates skeletal muscle atrophy in chronic kidney disease via AMPK/SIRT1/PGC-1alpha-mediated oxidative stress and mitochondrial dysfunction. Front Pharmacol.

[243] Luan P, D'Amico D, Andreux PA, Laurila PP, Wohlwend M, Li H, de Imamura T, Lima, Place N, Rinsch C, Zanou N, Auwerx J (2021). Urolithin a improves muscle function by inducing mitophagy in muscular dystrophy. Sci Transl Med..

[244] James R, Chaytow H, Ledahawsky LM, Gillingwater TH (2021). Revisiting the role of mitochondria in spinal muscular atrophy. Cell Mol Life Sci.

[245] Jeon SH, Choung SY (2021). Oyster hydrolysates attenuate muscle atrophy via regulating protein turnover and mitochondria biogenesis in C2C12 cell and immobilized mice. Nutrients.

[246] Wan Q, Zhang L, Huang Z, Zhang H, Gu J, Xu H, Yang X, Shen Y, Law BY, Zhu J, Sun H (2020). Aspirin alleviates denervation-induced muscle atrophy via regulating the Sirt1/PGC-1alpha axis and STAT3 signaling. Ann Transl Med.

[247] Ando S, Funato M, Ohuchi K, Inagaki S, Sato A, Seki J, Kawase C, Saito T, Nishio H, Nakamura S (2019). The protective effects of Levetiracetam on a human iPSCs-derived spinal muscular atrophy model. Neurochem Res.

[248] Enoki Y, Watanabe H, Arake R, Fujimura R, Ishiodori K, Imafuku T, Nishida K, Sugimoto R, Nagao S, Miyamura S (2017). Potential therapeutic interventions for chronic kidney disease-associated sarcopenia via indoxyl sulfate-induced mitochondrial dysfunction. J Cachexia Sarcopenia Muscle.

[249] Yeh CC, Liu HM, Lee MC, Leu YL, Chiang WH, Chang HH, Lee TY (2022). Phytochemical-rich herbal formula ATG-125 protects against sucrose-induced gastrocnemius muscle atrophy by rescuing Akt signaling and improving mitochondrial dysfunction in young adult mice. Mol Med Rep.

[250] Wu CS, Wei Q, Wang H, Kim DM, Balderas M, Wu G, Lawler J, Safe S, Guo S, Devaraj S (2020). Protective effects of ghrelin on fasting-induced muscle atrophy in aging mice. J Gerontol A Biol Sci Med Sci.

[251] Khalilov RA (2023). comprehensive review of advanced nano-biomaterials in regenerative medicine and drug delivery. Adv Biol Earth Sci.

[252] Chodari L, Dilsiz Aytemir M, Vahedi P, Alipour M, Vahed SZ, Khatibi SMH, Ahmadian E, Ardalan M, Eftekhari A (2021). Targeting mitochondrial biogenesis with polyphenol compounds. Oxid Med Cell Longev.

[253] Yang YX, Liu MS, Liu XJ, Zhang YC, Hu YY, Gao RS, Pang EK, Hou L, Wang JC, Fei WY (2022). Porous Se@SiO(2) nanoparticles improve oxidative injury to promote muscle regeneration via modulating mitochondria. Nanomedicine.

[254] Moschetti A, Dagda RK, Ryan RO (2021). Coenzyme Q nanodisks counteract the effect of statins on C2C12 myotubes. Nanomedicine.

[255] Liu Y, Liang J, Wang Q, He Y, Chen Y (2016). Copper nanoclusters trigger muscle cell apoptosis and atrophy in vitro and in vivo. J Appl Toxicol.

[256] Baar K, Wende AR, Jones TE, Marison M, Nolte LA, Chen M, Kelly DP, Holloszy JO (2002). Adaptations of skeletal muscle to exercise: rapid increase in the transcriptional coactivator PGC-1. FASEB J.

[257] Borges IBP, de Oliveira DS, Marie SKN, Lenario AM, Oba-Shinjo SM, Shinjo SK (2021). Exercise training attenuates ubiquitin-proteasome pathway and increases the genes related to autophagy on the skeletal muscle of patients with inflammatory myopathies. J Clin Rheumatol.

[258] Ebadi M, Bhanji RA, Mazurak VC, Montano-Loza AJ (2019). Sarcopenia in cirrhosis: from pathogenesis to interventions. J Gastroenterol.

[259] Broskey NT, Greggio C, Boss A, Boutant M, Dwyer A, Schlueter L, Hans D, Gremion G, Kreis R, Boesch C (2014). Skeletal muscle mitochondria in the elderly: effects of physical fitness and exercise training. J Clin Endocrinol Metab.

[260] Timmerman KL, Dhanani S, Glynn EL, Fry CS, Drummond MJ, Jennings K, Rasmussen BB, Volpi E (2012). A moderate acute increase in physical activity enhances nutritive flow and the muscle protein anabolic response to mixed nutrient intake in older adults. Am J Clin Nutr.

[261] Vargas-Ortiz K, Perez-Vazquez V, Diaz-Cisneros FJ, Figueroa A, Jimenez-Flores LM, Rodriguez-DelaRosa G, Macias MH (2015). Aerobic training increases expression levels of SIRT3 and PGC-1alpha in skeletal muscle of overweight adolescents without change in caloric intake. Pediatr Exerc Sci.

[262] Liu S, Yu C, Xie L, Niu Y, Fu L (2021). Aerobic exercise improves mitochondrial function in sarcopenia mice through Sestrin2 in an AMPKalpha2-dependent manner. J Gerontol A Biol Sci Med Sci.

[263] Konopka AR, Harber MP (2014). Skeletal muscle hypertrophy after aerobic exercise training. Exerc Sport Sci Rev.

[264] Memme JM, Erlich AT, Phukan G, Hood DA (2021). Exercise and mitochondrial health. J Physiol.

[265] Shen L, Meng X, Zhang Z, Wang T (2018). Physical exercise for muscle atrophy. Adv Exp Med Biol.

[266] Salucci S, Falcieri E (2020). Polyphenols and their potential role in preventing skeletal muscle atrophy. Nutr Res.

[267] Leduc-Gaudet JP, Hussain SNA, Barreiro E, Gouspillou G (2021). Mitochondrial dynamics and mitophagy in skeletal muscle health and aging. Int J Mol Sci.

[268] Wallace MA, Aguirre NW, Marcotte GR, Marshall AG, Baehr LM, Hughes DC, Hamilton KL, Roberts MN, Lopez-Dominguez JA, Miller BF (2021). The ketogenic diet preserves skeletal muscle with aging in mice. Aging Cell.

[269] Miller VJ, LaFountain RA, Barnhart E, Sapper TS, Short J, Arnold WD, Hyde PN, Crabtree CD, Kackley ML, Kraemer WJ (2020). A ketogenic diet combined with exercise alters mitochondrial function in human skeletal muscle while improving metabolic health. Am J Physiol Endocrinol Metab.

[270] Rong S, Wang L, Peng Z, Liao Y, Li D, Yang X, Nuessler AK, Liu L, Bao W, Yang W (2020). The mechanisms and treatments for sarcopenia: could exosomes be a perspective research strategy in the future?. J Cachexia Sarcopenia Muscle.

[271] Petrocelli JJ, Drummond MJ (2020). PGC-1alpha-targeted therapeutic approaches to enhance muscle recovery in aging. Int J Environ Res Public Health.

[272] Tsien C, Davuluri G, Singh D, Allawy A, Ten Have GA, Thapaliya S, Schulze JM, Barnes D, McCullough AJ, Engelen MP (2015). Metabolic and molecular responses to leucine-enriched branched chain amino acid supplementation in the skeletal muscle of alcoholic cirrhosis. Hepatology.

[273] Cannavino J, Brocca L, Sandri M, Bottinelli R, Pellegrino MA (2014). PGC1-alpha over-expression prevents metabolic alterations and soleus muscle atrophy in hindlimb unloaded mice. J Physiol.

[274] Theilen NT, Jeremic N, Weber GJ, Tyagi SC (2019). TFAM overexpression diminishes skeletal muscle atrophy after hindlimb suspension in mice. Arch Biochem Biophys.

[275] Leduc-Gaudet JP, Mayaki D, Reynaud O, Broering FE, Chaffer TJ, Hussain SNA, Gouspillou G (2020). Parkin overexpression attenuates sepsis-induced muscle wasting. Cells.

[276] Chalkiadaki A, Igarashi M, Nasamu AS, Knezevic J, Guarente L (2014). Muscle-specific SIRT1 gain-of-function increases slow-twitch fibers and ameliorates pathophysiology in a mouse model of duchenne muscular dystrophy. PLoS Genet.

[277] Goljanek-Whysall K, Soriano-Arroquia A, McCormick R, Chinda C, McDonagh B (2020). miR-181a regulates p62/SQSTM1, parkin, and protein DJ-1 promoting mitochondrial dynamics in skeletal muscle aging. Aging Cell.

[278] Cai B, Ma M, Zhang J, Wang Z, Kong S, Zhou Z, Lian L, Zhang J, Li J, Wang Y (2022). LncEDCH1 improves mitochondrial function to reduce muscle atrophy by interacting with SERCA2. Mol Ther Nucleic Acids.

[279] Alessio E, Buson L, Chemello F, Peggion C, Grespi F, Martini P, Massimino ML, Pacchioni B, Millino C, Romualdi C (2019). Single cell analysis reveals the involvement of the long non-coding RNA Pvt1 in the modulation of muscle atrophy and mitochondrial network. Nucl Acids Res.

[280] Yu J, Loh K, Yang HQ, Du MR, Wu YX, Liao ZY, Guo A, Yang YF, Chen B, Zhao YX (2022). The Whole-transcriptome landscape of diabetes-related sarcopenia reveals the specific function of novel lncRNA Gm20743. Commun Biol.

[281] Piao L, Huang Z, Inoue A, Kuzuya M, Cheng XW (2022). Human umbilical cord-derived mesenchymal stromal cells ameliorate aging-associated skeletal muscle atrophy and dysfunction by modulating apoptosis and mitochondrial damage in SAMP10 mice. Stem Cell Res Ther.

[282] Lo JH, Yiu UKP, Ong MT, Lee WY (2020). Sarcopenia: Current treatments and new regenerative therapeutic approaches. J Orthop Translat.

[283] Hafen PS, Abbott K, Bowden J, Lopiano R, Hancock CR, Hyldahl RD (1985). Daily heat treatment maintains mitochondrial function and attenuates atrophy in human skeletal muscle subjected to immobilization. J Appl Physiol.

[284] Guigni BA, Fix DK, Bivona JJ, Palmer BM, Carson JA, Toth MJ (2019). Electrical stimulation prevents doxorubicin-induced atrophy and mitochondrial loss in cultured myotubes. Am J Physiol Cell Physiol.

[285] Ou HC, Chu PM, Huang YT, Cheng HC, Chou WC, Yang HL, Chen HI, Tsai KL (2021). Low-level laser prevents doxorubicin-induced skeletal muscle atrophy by modulating AMPK/SIRT1/PCG-1alpha-mediated mitochondrial function, apoptosis and up-regulation of pro-inflammatory responses. Cell Biosci.

[286] Xie WQ, Xiao WF, Tang K, Wu YX, Hu PW, Li YS, Duan Y, Lv S (2020). Caloric restriction: implications for sarcopenia and potential mechanisms. Aging.

